# Supplementary material for: Systematics of Ditaxinae and Related Lineages within the Subfamily Acalyphoideae (Euphorbiaceae) Based on Molecular Phylogenetics
Source: Biology (Basel). 2023 Jan 21;12(2):173. doi: 10.3390/biology12020173 (PMC9952443; doi:10.3390/biology12020173)
Supplement: Supplementary file 1 [file biology-12-00173-s001.zip › Supplementary material/Supplementary File S1.pdf]

SUPPLEMENTARY MATERIAL S1

Systematics of Ditaxinae and related lineages within subfamily Acalyphoideae (Euphorbiaceae) based on molecular phylogenetics

Josimar Külkamp, Ricarda Riina, Yocupitzia Ramírez-Amezcua, João R.V. Iganci, Inês Cordeiro, Raquel González-Páramo, Sabina Irene Lara-Cabrera and José Fernando A. Baumgratz

Supplementary material S1: Supplementary table.

TABLE S1: Taxa, voucher information and accessions used in this study. GenBank's access numbers are presented in the columns of the molecular markers. The names of the taxa follow the topology of the trees, for the updated names, see the "taxonomic treatment" section of the manuscript.

Taxa	Samples precedence	DNA code	Collector	Country	Locality	ITS	ETS	*pet*D	*trn*LF	*trn*TL
Acalypha lanceolata Willd.	Genbank	SamKurukura	S. Kurukura & A Hassan C6_K1342	Kenya		MK261283.1			MK261314.1	
Acalypha lanceolata Willd.	Genbank	DinhDuyVu	Dinh Duy Vu et al. TSL07	Vietnam		MZ505521.1			AY794762.1	
Adelia brandegeei V.W.Steinm.	Silica	YPR1510	Y. Ramírez-Amezcua, V.W. Steinmann & Y. Yang 1510 (IEB)	Mexico	Baja California Sur. Municipio de Los Cabos	OQ190564			OQ151648	
Adelia cinerea (Wiggins & Rollins) A. Cerv., V.W. Steinm. & Flores Olv.	Genbank	Steinmann971	V. Steinmann 971 (MEXU)	Mexico				HG97197.2	HG97180.5	

Adelia oaxacana (Müll.Arg.) Hemsl.	Silica	Stei2974	V.W. Steinmann 2974 (IEB)	Mexico	Michoacán. Municipio de La Huacana	OQ190565			OQ151649	
Adelia sp.	Herbarium	rr673	M. Nee 30013 (MA)	Mexico	Veracruz, Mun. Hidalgotitlan	OQ190567	OQ183622		OQ151650	OQ116469
Adelia triloba (Müll.Arg.) Hemsl.	Herbarium	rr674	R. Espinoza 1676 (MA)	Costa Rica	Guanacaste, Liberia	OQ190566	OQ183621			
Argythamnia candicans Sw.	Genbank	Gillis12922	W.T. Gillis 12922 (B)	Bahamas				HG97198.4	HG97181.7	
Argythamnia candicans Sw.	Genbank	GBBorsch5122	T. Borsch et al. 5122 (B)	Cuba				HG97198.5	HG97181.8	
Argythamnia candicans Sw.	Herbarium	78	Y. Ramírez-Amezcua et al. 1942 (IEB)	Cuba	Santiago	OQ190570				
Argythamnia candicans Sw.	Herbarium	79	Y. Ramírez-Amezcua et al. 1941 (IEB)	Cuba	Gibara	OQ190571				
Argythamnia candicans Sw.	Herbarium	80	Y. Ramírez-Amezcua et al. 1936 (IEB)	Cuba	Cienfuegos	OQ190572				
Argythamnia candicans Sw.	Herbarium	YPRPR	F. Axelrod et al. 2554 (NY)	Puerto Rico	Isabella	OQ190569				
Argythamnia candicans Sw.	Herbarium	YPR	F. Axelrod et al. 2554 (NY)	Puerto Rico	Isabella	OQ190573			OQ151652	
Argythamnia coatepensis (Brandegee) Croizat	Silica	rr579	G.J. Garcia & C.A.C. Espinosa 624 (NY)	México	Oaxaca	OQ190574	OQ183624		OQ151653 OQ151654	
Argythamnia cubensis Britton & P.Wilson	Silica	YPR1943	Y. Ramírez-Amezcua, J.A. Pérez-Camacho & M. Cañizares 1943 (IEB)	Cuba	Provincia de Santiago de Cuba	OQ190575			OQ151655	
Argythamnia ecdyomena J.W.Ingram	Silica	rr580	B. Wallnofer & F.M. Tut-Tesucum 5940 (NY)	Guatemala	Petén, San Andrés	OQ190576	OQ183625		OQ151656	

Argythamnia ecdyomena J.W.Ingram	Silica	YPR5940	B. Wallnofer & F.M. Tut-Tesucum 5940 (1) (NY)	Guatemala	Petén, San Andrés				OQ151657	
Argythamnia lottiae J.W.Ingram	Herbarium	rr581	Y. Ramírez-Amezcua et al. 807 (IEB)	México	Jalisco, La Huerta, Manzanilla	OQ190577	OQ183626		OQ151658	
Argythamnia lottiae J.W.Ingram	Silica	y100	Y. Ramírez-Amezcua et al. 807 (1) (IEB)	Mexico	Michoacán	OQ190578				
Argythamnia lottiae J.W.Ingram	Herbarium	y144	Y. Ramírez-Amezcua & V.W. Steinmann 1893 (IEB)	Mexico	Guerrero	OQ190579			OQ151659	
Argythamnia lucayana Millsp.	Herbarium	rr582	D.S. Correll 44052 (NY)	Bahamas	Exuma, Gran Exuma	OQ190580	OQ183627		OQ151660 OQ151661 OQ151662	
Argythamnia lucayana Millsp.	Herbarium	y83	D.S. Correll 50829 (NY)	Bahama Islands	Great Exuma	OQ190581				OQ116470
Argythamnia lucayana Millsp.	Herbarium	YPR	D.S. Correll 50829 (1) (NY)	Bahama Islands	Great Exuma	OQ190582			OQ151663	
Argythamnia lundellii J.W.Ingram	Herbarium	rr583	Y. Ramírez-Amezcua et al. 2033 (MEXU)	México	Yucatán, Chichén Itzá	OQ190583	OQ183628		OQ151664 OQ151665	
Argythamnia lundellii J.W.Ingram	Herbarium	y96	Cabrera 13051 (XAL)	México	Quintana Roo	OQ190584			OQ151666	
Argythamnia moorei J.W.Ingram	Herbarium	rr584	D.E. Breedlove 69797 (CAS)	México	Chiapas	OQ190585	OQ183629		OQ151667	OQ116471
Argythamnia moorei J.W.Ingram	Herbarium	YPR	D.E. Breedlove 69797 (1) (CAS)	México	Chiapas	OQ190586			OQ151668	
Argythamnia sericea Griseb.	Herbarium	rr585	D.S. Correll & G.R. Proctor 48837 (US)	Bahamas	Long Cay, Crooked Island	OQ190587 OQ190588	OQ183630		OQ151669 OQ151670	
Argythamnia sericea Griseb.	Herbarium	YPR	D.S. Correll & G.R. Proctor 48837 (1) (US)	Bahamas	Long Cay, Crooked Island	OQ190589			OQ151671	

Argythamnia sp.	Herbarium	rr586	V.W. Steinmann s.n. (IEB)	USA	St. Croix Island	OQ190590	OQ18363 1		OQ151672 OQ151673	OQ116472
Argythamnia sp.	Genbank	Lott2391	E. Lott 2391 (MEXU)	Mexico				HG97203.4	HG97186.7	
Argythamnia sp.	Sílica	StCrYPR	V.W. Steinmann s.n. (IEB)	USA	St. Croix Island	OQ190591			OQ151674	
Argythamnia tinctoria Millsp.	Silica	rr587	E. Contreras 10383 (US)	Guatemal a	Petén, San Francisco, San Juan	OQ190592	OQ18363 3		OQ151675	
Argythamnia wheeleri J.W.Ingram	Herbarium	YPR	J.C. Trejo et al. 559 (XAL)	México	Yucatán	OQ190593			OQ151676	
Bernardia dichotoma (Willd.) Müll.Arg.	Genbank	Jestrow1001	B. Jestrow 1001 (FTG)	Jamaica		GU000029.1			EF470582.1	
Bernardia dichotoma (Willd.) Müll.Arg.	Genbank	Clase5374	T. Clase et al. 5374 (JBSD)	Dominica n Republic					HG971822.1	
Caperonia bahiensis Müll.Arg.	Silica	rr588	M. Arbo 8731 (BA)	Argentina	Corrientes, Corrientes				OQ151677	
Caperonia castaneifolia (L.) A.St.-Hil.	Silica	rr590	J. Külkamp 778 (RB)	Brasil	Río de Janeiro, Squarema	OQ190594			OQ151678	OQ116521
Caperonia castaneifolia (L.) A.St.-Hil.	Silica	rr591	J. Külkamp 1042 (RB)	Brasil	Espírito Santo, Sooretama, Lagoa Juparana		OQ18363 5		OQ151679	
Caperonia chiltepecensis Croizat	Silica	rr593	R.L. Luna 557 (NY)	Mexico	Oaxaca	OQ190595			OQ151680	
Caperonia corchoroides Müll.Arg.	Silica	rr594	D. Zappi et al. 1458 (NY)	Brasil	Mato Grosso, Nuevo Mundo	OQ190596			OQ151681	
Caperonia cordata A.St.-Hil.	Silica	rr592	J. Schaefer & S.A. Bordignon s.n. (ICN-193858)	Brasil	Río Grande do Sul, Unistalda, Porteirinha	OQ190712	OQ18363 6	OQ151622	OQ151682	OQ116522
Caperonia cordata A.St.-Hil.	Silica	rr595	Aliscioni et al. 584 (SI)	Argentina	Formosa, Formosa				OQ151683	

Caperonia cubana Pax & K.Hoffm.	Genbank	Greuter25237	W. Greuter et al. 25237 (B)	Cuba				HG97199.8	HG97183.1	
Caperonia fistulosa Beille	Herbarium	IS2024	De Witte 5782 (MO)	Congo		OQ190724				
Caperonia heteropetala Didr.	Silica	rr596	J. Külkamp 1028 (RB)	Brasil	Espírito Santo, Linhares, Vale do Suruaca	OQ190713		OQ151623	OQ151684	OQ116523
Caperonia heteropetala Didr.	Silica	rr597	J. Külkamp 1043 (RB)	Brasil	Espírito Santo, Sooretama, Lagoa Juparana	OQ190715		OQ151624	OQ151685	OQ116524
Caperonia heteropetala Didr.	Silica	rr598	J. Külkamp 1240 (RB)	Brasil	Río de Janeiro, Silva Jardim	OQ190716			OQ151686	OQ116525
Caperonia sp.	Silica	rr599	E. Valduga et al. 788 (ICN)	Brasil	Río Grande do Sul, Torres, Itapeva	OQ190717	OQ183637		OQ151687	OQ116526
Caperonia palustris (L.) A.St.-Hil.	Silica	rr601	J. Külkamp 515 (RB)	Brasil	Mato Grosso do Sul, Corumbá	OQ190718 OQ190719	OQ183638	OQ151625	OQ151688	OQ116527
Caperonia palustris (L.) A.St.-Hil.	Silica	rr602	J. Külkamp 511 (RB)	Brasil	Mato Grosso do Sul, Guia Lopes da Laguna	OQ190714	OQ183641	OQ151626	OQ151692	OQ116531
Caperonia palustris (L.) A.St.-Hil.	Genbank	HG9719991	T. Borsch et al. 4389 (B)	Cuba				HG971999.1	HG97183.2	
Caperonia palustris (L.) A.St.-Hil.	Genbank	GBWurdackD073	K. Wurdack D073 (US)			MK780933.1			AY794745.1	
Caperonia serrata (Turcz.) C.Presl.	Herbarium	IS2020	Jongkind 2468 (MO)	Ghana		OQ190723				
Caperonia serrata (Turcz.) C.Presl.	Herbarium	IS2023	Mahlangu 607 (MO)	Zimbabwe		OQ190720	OQ183639		OQ151689	OQ116528
Caperonia sp.	Herbarium	IS2027	Nsiowndele 2436 (MO)	Congo		OQ190721	OQ183640		OQ151690	OQ116529

Caperonia sp.	Herbarium	IS2028	Kuchar 23087 (MO)	Tanzania		OQ190722			OQ151691	OQ116530
Caperonia stuhlmannii Pax	Herbarium	IS2026	Kayombo 2307 (MO)	Tanzania		OQ190725			OQ151693	OQ116532
Caperonia stuhlmannii Pax	Genbank	GBJG16P06A2	J. Guyton & B. Wursten s.n. (no collection cited)	Mozambi que	Sofala, Gorongosa, Gorongos, National Park, Lion House				MK186998.1	
Caperonia stuhlmannii Pax	Genbank	GBJG16P01E9	J. Guyton & B. Wursten (1) s.n. (no collection cited)	Mozambi que	Sofala, Gorongosa, Gorongos, National Park, Lion House				MK186999.1	
Chiropetalum anisotrichum (Müll.Arg.) Pax & K.Hoffm.	Silica	rr604	J. Külkamp et al. 210 (HUEFS)	Brasil	Santa Catarina, Lauro Müller	OQ190599	OQ18364 2	OQ151627	OQ151696	OQ116474
Chiropetalum anisotrichum (Müll.Arg.) Pax & K.Hoffm.	Silica	rr605	J. Külkamp et al. 148 (ICN)	Brasil	Santa Catarina, Florianópolis	OQ190600	OQ18364 3	OQ151628	OQ151697	OQ116475
Chiropetalum anisotrichum (Müll.Arg.) Pax & K.Hoffm.	Külkamp-Masters	K3	J. Külkamp et al. 222 (ICN)	Brasil	Rio Grande do Sul, Pareci Novo	OQ190597			OQ151694	
Chiropetalum anisotrichum (Müll.Arg.) Pax & K.Hoffm.	Külkamp-Masters	K4	J. Külkamp et al.148 (ICN)	Brasil	Santa Catarina, Florianópolis	OQ190598			OQ151695	
Chiropetalum argentinense Skottsb.	Silica	rr606	L. Carbone 736 (ACOR)	Argentina	Córdoba, Córdoba capital	OQ190601	OQ18364 4	OQ151629	OQ151698	OQ116473
Chiropetalum argentinense Skottsb.	Silica	rr612	J. Külkamp 1248 (BA)	Argentina	Córdoba, Reserva General San Martín	OQ190602	OQ18364 5	OQ151630	OQ151699	OQ116476

Chiropetalum astroplethos (J.W.Ingram) Radcl.-Sm. & Govaerts	Herbarium	YPRY7	Hinton et al. 21091 (IEB)	Mexico	Coahuila. Ramos Arizpe				OQ151700	
Chiropetalum berterianum Schltdl.	Herbarium	YPR	L.R. Landrum et al. 10059 (NY)	Chile	Region Met, Santiago				OQ151701	
Chiropetalum berterianum var. *psiladenium* Skottsb.	Herbarium	rr607	O. Zollner 8248 (NY)	Chile	Coquimbo, Guanaqueros, near of coast	OQ190603	OQ18364 6	OQ151631	OQ151702	OQ116477
Chiropetalum boliviense (Müll.Arg.) Pax & K.Hoffm.	Herbarium	YPRTR	M. Nee et al. 52061 (NY)	Bolivia	Depto Santa Cruz. Prov. Florida, Parque Nacional Amboró	OQ190604			OQ151703	
Chiropetalum canescens (Ph il.) F.Phil.	Herbarium	rr609	S. Teillier 608 (NY)	Chile	Antofagasta, Antofagasta	OQ190605	OQ18364 7		OQ151704	OQ116478
Chiropetalum canescens (Ph il.) F.Phil.	Herbarium	y82	S. Teillier 608 (1) (NY)	Chile	Antofagasta, Antofagasta	OQ190606				
Chiropetalum foliosum Pax & K.Hoffm	Silica	rr610	J. Külkamp et al. 230 (ICN)	Brasil	Río Grande do Sul, Porto Alegre	OQ190608	OQ18364 8	OQ151632	OQ151706	OQ116479
Chiropetalum foliosum Pax & K.Hoffm	Külkamp-Masters	K1	J. Külkamp et al. 231 (ICN)	Brasil	Rio Grande do Sul, Porto Alegre	OQ190607			OQ151705	
Chiropetalum griseum Griseb.	Silica	rr611	J. Külkamp 1249 (BA)	Argentina	Córdoba, Reserva General San Martín	OQ190609	OQ18364 9	OQ151633	OQ151707	OQ116480
Chiropetalum griseum Griseb.	Silica	rr613	M.P. Suarez Santillán 1212 (ACOR)	Argentina	Córdoba, Pocho	OQ190610	OQ18365 0	OQ151634	OQ151708	OQ116481
Chiropetalum griseum Griseb.	Herbarium	YPR54	Krapovickas et al. 27058 (MO)	Argentina	Corrientes, Corrientes	OQ190611				

Chiropetalum intermedium Pax & K.Hoffm.	Silica	rr614	J. Külkamp et al. 154 (ICN)	Brasil	Río Grande do Sul, Caçapava do Sul	OQ190614	OQ18365 1	OQ151635	OQ151711	OQ116483
Chiropetalum intermedium Pax & K.Hoffm.	Külkamp-Masters	K2	J. Külkamp et al. 74 (ICN)	Brasil	Río Grande do Sul, Caçapava do Sul	OQ190613			OQ151710	
Chiropetalum latifolium (Chodat & Hassl.) Külkamp	Silica	rr615	J. Külkamp et al. 441 (ICN)	Brasil	Río Grande do Sul, Derrubadas	OQ190615	OQ18365 2	OQ151636	OQ151712	OQ116484
Chiropetalum latifolium (Chodat & Hassl.) Külkamp	Silica	rr616	J. Külkamp et al. 365 (ICN)	Brasil	Río Grande do Sul, Quaraí	OQ190616	OQ18365 3	OQ151637	OQ151713	OQ116485
Chiropetalum latifolium (Chodat & Hassl.) Külkamp	Herbarium	rr617	Ortiz & E. Zardini 672 (MO)	Paraguay	Paraguarí, Mbatovi Santo Tomás	OQ190617	OQ18365 4	OQ151638	OQ151714	
Chiropetalum latifolium (Chodat & Hassl.) Külkamp	Herbarium	y80	E. Zardini et al. 12788 (MO)	Paraguay	Paraguarí		OQ18365 9			
Chiropetalum latifolium (Chodat & Hassl.) Külkamp	Genbank	GBZardini30777	E. Zardini & T. Tilleria 30777 (B)	Paraguay				HG97200.5	HG97183.8	
Chiropetalum latifolium (Chodat & Hassl.) Külkamp	Herbarium	y89	Ortiz & E. Zardini 672 (MO)	Paraguay		OQ190634	OQ18366 3			OQ116493
Chiropetalum latifolium (Chodat & Hassl.) Külkamp	Herbarium	YPR	Ortiz & E. Zardini 672 (1) (MO)	Paraguay		OQ190635				
Chiropetalum molle (Klotzsch ex. Baill.) Pax & K.Hoffm.	Silica	rr618	J. Külkamp et al. 207 (ICN)	Brasil	Santa Catarina, Urupema	OQ190620	OQ18365 5	OQ151639	OQ151717	OQ116486
Chiropetalum molle (Klotzsch ex. Baill.) Pax & K.Hoffm.	Külkamp-Masters	K5	A. Melo & M. Grins s.n. (ICN-197014)	Brasil	Rio Grande do Sul, Bagé	OQ190618			OQ151715	
Chiropetalum molle (Klotzsch ex. Baill.) Pax & K.Hoffm.	Külkamp-Masters	K6	J. Külkamp et al. 208 (ICN)	Brasil	Santa Catarina, Urupema	OQ190619			OQ151716	
Chiropetalum phalacradenium	Silica	rr619	J. Külkamp et al. 1315 (RB)	Brasil	Santa Catarina,	OQ190622	OQ18365 6	OQ151640	OQ151719	OQ116487

(J.W.Ingram) L.B.Sm. & Downs					Anitápolis, Trilha dos Índios					
Chiropetalum phalacradenium (J.W.Ingram) L.B.Sm. & Downs	Külkamp-Masters	K7	J. Külkamp et al. 216 (ICN)	Brasil	Santa Catarina, Lauro Müller, Serra do Rio do Rastro	OQ190621			OQ151718	
Chiropetalum puntaloberense Alonso Paz & Bassagoda	Silica	rr620	J. Külkamp 801 (RB)	Uruguay	Rocha, La Coronilla	OQ190623	OQ18365 7	OQ151641	OQ151721	OQ116488
Chiropetalum quinquecuspidatum (A.Juss.) Pax & K.Hoffm.	Silica	rr621	L. Hoogte & C. Roersch 3534 (NY)	Perú	Puno, Sandia	OQ190624	OQ18366 7	OQ151642	OQ151722	OQ116489
Chiropetalum ramboi (Allem & Irgang) Radcl.-Sm. & Govaerts	Silica	rr622	J. Carrion 1719 (HUEFS)	Brasil	Río Grande do Sul, Torres	OQ190626	OQ18365 8	OQ151643	OQ151724	OQ116490
Chiropetalum ramboi (Allem & Irgang) Radcl.-Sm. & Govaerts	Külkamp-Masters	K8	J. Külkamp et al. 209 (ICN)	Brasil	Río Grande do Sul, Torres	OQ190625			OQ151723	
Chiropetalum schiedeanum (Müll.Arg.) Pax	Silica	rr623	Y. Ramirez-Amezcua et al. 1038 (NY)	México	Morelos	OQ190628	OQ18366 0	OQ151644	OQ151726	OQ116491
Chiropetalum schiedeanum (Müll.Arg.) Pax	Silica	YPRY2	Y. Ramirez-Amezcua et al. 1038 (1) (NY)	México	Morelos	OQ190627			OQ151725	
Chiropetalum schiedeanum (Müll.Arg.) Pax	Silica	rr624	G.S. Hinton 22645 (NY)	México	Puebla, Zaragoza	OQ190629	OQ18366 1	OQ151645	OQ151727	
Chiropetalum schiedeanum (Müll.Arg.) Pax	Genbank	GBCarranza3565	Carranza 3565 (IEB)	México					AY794744.1	
Chiropetalum sp.	Herbarium	rr625	O. Fernandes 195 (JBN)	Chile	Valparaiso, Viña del Mar, Cultivanda no	OQ190632		OQ151646	OQ151729	

					Jardim Botânico Nacional					
Chiropetalum sp.	Herbarium	PnovoaYPR	O. Fernandes 195 (1) (IEB)	Chile	Valparaiso, Viña del Mar, Cultivanda no Jardim Botânico Nacional	OQ190630			OQ151720	
Chiropetalum sp.	Genbank	GBWeigend5670	M. Weigend et al. 5670 (B)	Peru				HG97200.4	HG97183.7	
Chiropetalum tricoccum (Vell.) Chodat & Hassl	Herbarium	YPRNY	Jardim et al. 1498 (NY)	Brasil	Bahia	OQ190612			OQ151709	
Chiropetalum tricoccum (Vell.) Chodat & Hassl	Genbank	GBDittrich193	Dittrich 193 (NY)	Brasil	Paraná				AY794743.1	
Chiropetalum tricoccum (Vell.) Chodat & Hassl	Silica	rr603	L.P. Santos 322 (SP)	Brasil	São Paulo	OQ190631	OQ183662		OQ151728	OQ116492
Chiropetalum tricoccum (Vell.) Chodat & Hassl	Silica	rr626	J. Külkamp et al. 615 (RB)	Brasil	Bahía, Jussari	OQ190633	OQ183664		OQ151730	
Chiropetalum tricuspidatum (Lam.) A.Juss.	Herbarium	y93	E.J. Tepe & P.B. Pelser 2036 (RSA)	Chile	Bío-Bío, Concepción (Región VIII)	OQ190636	OQ183665		OQ151731	OQ116482
Chiropetalum tricuspidatum (Lam.) A.Juss.	Herbarium	rr627	E.J. Tepe & P.B. Pelser 2036 (1) (RSA)	Chile	Bío-Bío, Concepción (Región VIII)		OQ183666			
Ditaxis acutangula	Herbarium	YPR	H. Mendoza et al. 9 (COL)	Colombia	Tolima				OQ151733	
Ditaxis adenophora (A.Gray) Pax & K.Hoffm.	Herbarium	y132	R. Guerrero & Van Devender 2010-37 (IEB)	México	Sonora	OQ190639	OQ183672			
Ditaxis adenophora (A.Gray) Pax & K.Hoffm.	Herbarium	rr630	I.L. Wiggins & C.R. Rollins 183 (US)	México	Sonora, Guaymas, San Carlos	OQ190638	OQ183669		OQ151734	

Ditaxis adenophora (A.Gray) Pax & K.Hoffm.	Herbarium	YPR	P. Fryxell 3078 (MICH)	México	Sonora, 30 miles S of Benjamín Hill				OQ151735	
Ditaxis aphoroides (Müll.Arg.) Pax	Herbarium	y94	W.R. Carr 18104 (IEB)	USA	Texas	OQ190642			OQ151738	
Ditaxis argothamnoides (Bertero ex Spreng.) Radcl.-Sm. & Govaerts	Genbank	GBWurdackD105	K. Wurdack D105 (US)	USA		MK780949.1	MK781080.1		AY794741.1	
Ditaxis argyraea Cory	Herbarium	rr578	H.B. Parks 24134 (F)	USA	Texas	OQ190568	OQ183623		OQ151651	
Ditaxis argyraea Cory	Herbarium	YPR	H.B. Parks 24134 (1) (F)	USA	Texas				OQ151739	
Ditaxis blodgettii (Torr. ex Chapm.) Pax	Herbarium	rr635	D.S. Correll & J. Popenoe 48121 (NY)	USA	Florida, Miami-Dade, The Hammocks	OQ190644	OQ183676		OQ151741	
Ditaxis blodgettii (Torr. ex Chapm.) Pax	Herbarium	YPR4	D.S. Correll & J. Popenoe 48121 (1) (NY)	USA	Florida, Miami-Dade, The Hammocks	OQ190643			OQ151740	
Ditaxis brandegeei var. *brandegeei* (Millsp.) Rose & Standl.	Herbarium	branYPR	Y. Ramirez-Amezcua et al. 1528 (IEB)	México	Baja California Sur.	OQ190645			OQ151742	
Ditaxis brandegeei var. *brandegeei* (Millsp.) Rose & Standl.	Herbarium	YPRMF31	M. Fishbein et al. 3113 (IEB)	Mexico	Baja California Sur. Sierra La Giganta				OQ151743	

Ditaxis brandegeei var. *intosa* I.M.Johnst.	Herbarium	rr636	Y. Ramirez-Amezcua et al. 1529 (IEB)	México	Baja California, Loreto	OQ190647	OQ183677		OQ151744	OQ116494
Ditaxis brandegeei var. *intosa* I.M.Johnst.	Herbarium	YPRTR	Y. Ramirez-Amezcua et al. 1529 (1) (IEB)	México	Baja California, Loreto	OQ190646			OQ151745	
Ditaxis breviramea (Müll.Arg.) Pax & K.Hoffm.	Silica	rr637	J. Külkamp 563 (RB)	Argentina	Jujuy	OQ190648	OQ183678	OQ151647	OQ151747	OQ116495
Ditaxis breviramea (Müll.Arg.) Pax & K.Hoffm.	Herbarium	YPR48	M. Nee & E. Chávez 48919 (NY)	Bolivia	Depto Santa Cruz. Prov. Florida	OQ190650			OQ151746	
Ditaxis breviramea (Müll.Arg.) Pax & K.Hoffm.	Herbarium	YPR92	M. Nee & E. Chávez 48919 (1) (NY)	Bolivia	Depto Santa Cruz. Prov. Florida	OQ190649				
Ditaxis claryana (Jeps.) G.L.Webster	Herbarium	YPR3384	S.J. de Groot & N. Fraga 3384 (RSA)	USA	San Bernardino Co., Whipple Mountains	OQ190653				
Ditaxis claryana (Jeps.) G.L.Webster	Herbarium	YPR	S.J. de Groot & N. Fraga 3384 (1) (RSA)	USA	San Bernardino Co., Whipple Mountains	OQ190655			OQ151749	
Ditaxis claryana (Jeps.) G.L.Webster	Herbarium	YPR94	S.J. De Groot & N. Fraga 94 (F)	USA	San Bernardino	OQ190654				
Ditaxis cyanophylla Wooton & Standl.	Herbarium	rr638	D. Thornburg 597 (NY)	USA	Arinona	OQ190656	OQ183680		OQ151750	OQ116498
Ditaxis cyanophylla Wooton & Standl.	Herbarium	YPR9165	M.A. Becker 9165 (RSA)	USA	Arinona	OQ190657				
Ditaxis desertorum (Müll.Arg.) Pax & K.Hoffm.	Silica	rr639	J. Külkamp et al. 631 (RB)	Brasil	Bahía, Iaçu	OQ190658	OQ183681		OQ151751	OQ116496
Ditaxis desertorum (Müll.Arg.) Pax & K.Hoffm.	Silica	rr640	J. Külkamp et al. 703 (RB)	Brasil	Bahía, Ipirá	OQ190659	OQ183682		OQ151752	OQ116497

Ditaxis dioica Kunth	Silica	rr641	L.R. Quiroz 1760 (NY)	Perú	Cajamarca, Jaén	OQ190662	OQ18367 1			
Ditaxis dioica Kunth	Herbarium	y92	H. Van der Werff, R. Vasquez & B. Gray 16411 (MOL)	Perú	Cajamarca, Jaén	OQ190709	OQ18363 2			
Ditaxis dioica Kunth	Herbarium	rr669	H. Van der Werff, R. Vasquez & B. Gray 16411 (MOL)	Perú	Cajamarca, Jaén	OQ190708			OQ151800	
Ditaxis dioica Kunth	Herbarium	MOLYPR	H. Van der Werff, R. Vasquez & B. Gray 16411 (1) (MOL)	Perú	Cajamarca, Jaén; 05.42S, 078.48W. 25 Mar. 2001				OQ151801	
Ditaxis fasciculata (Vahl ex A. Juss.) Müll.Arg.	Herbarium	rr642	P. Acevedo-Rodriguez & A. Siaca 3866 (US)	USA	Virgin Islands, St. John, Coral Bay	OQ190663	OQ1836 83		OQ151753	
Ditaxis fasciculata (Vahl ex A. Juss.) Müll.Arg.	Herbarium	y81	P. Acevedo-Rodriguez & A. Siaca 3866 (1) (US)	USA	Virgin Islands, St. John, Coral Bay	OQ190665	OQ18368 5		OQ151755	
Ditaxis fasciculata (Vahl ex A. Juss.) Müll.Arg.	Silica	rr643	P. Acevedo-Rodriguez et al. 2562 (NY)	USA	Virgin Islands, Central, Saint John	OQ190664	OQ18368 4		OQ151754	OQ116502
Ditaxis grazielae Külkamp	Silica	rr644	J. Külkamp et al. 689 (RB)	Brasil	Bahía, Jussara	OQ190666	OQ18368 6		OQ151756	
Ditaxis grazielae Külkamp	Silica	rr645	J. Külkamp et al. 688 (RB)	Brasil	Bahía, Jussara	OQ190667	OQ18368 7		OQ151757	OQ116505
Ditaxis guatemalensis (Müll.Arg.) Pax & K.Hoffm.	Silica	rr646	Y. Ramirez-Amezcua 1094 (NY)	México	Querétaro, El Marqués	OQ190668	OQ18368 8		OQ151758	OQ116507
Ditaxis guatemalensis (Müll.Arg.) Pax & K.Hoffm.	Genbank	GBGonzalez6363	S. González & R. R. Clinebell 6363 (MEXU)	Mexico				HG97203.0	HG97186.3	
Ditaxis guatemalensis (Müll.Arg.) Pax & K.Hoffm.	Silica	YPRVWS7	V.W. Steinmann 723 (IEB)	Mexico		DQ997792.1			OQ151759	

Ditaxis guatemalensis (Müll.Arg.) Pax & K.Hoffm.	Herbarium	YPR	V.W. Steinmann & Y. Ramírez-Amezcua, 5538 (IEB)	Mexico	Michoacán. Municipio de Múgica	OQ190669			OQ151760	
Ditaxis guatemalensis (Müll.Arg.) Pax & K.Hoffm.	Genbank	GBVentura7224	E. Ventura & E. Lopez 7224 (MEXU)	Mexico				HG972031.1	HG971864.1	
Ditaxis heterantha Zucc.	Silica	rr647	Y. Ramirez-Amezcua 638 (NY)	México	Michoacán, Jiquilpan	OQ190670	OQ183689		OQ151762	OQ116508
Ditaxis heterantha Zucc.	Silica	YPRTR	Y. Ramirez-Amezcua 638 (1) (NY)	México	Michoacán, Jiquilpan	OQ190671			OQ151761	
Ditaxis humilis (Engelm. & A.Gray) Pax	Herbarium	rr648	M. Nee 57-75 (NY)	USA	Texas	OQ190672	OQ183690		OQ151763	OQ116509
Ditaxis humilis (Engelm. & A.Gray) Pax	Herbarium	YPR	T.R. Van Devender 96-295 (RSA)	USA	Texas	OQ190673				
Ditaxis jablonszkyana (Pax & K.Hoffm.) J.W.Ingram	Silica	rr650	J. Külkamp 1247 (BA)	Argentina	San Juan, Marayes	OQ190674	OQ183691		OQ151764	OQ116512
Ditaxis jablonszkyana (Pax & K.Hoffm.) J.W.Ingram	Herbarium	YPR	A.T. Hunziker 22428 (F)	Argentina	General Lavalle				OQ151765	
Ditaxis lanceolata (Benth.) Pax & K.Hoffm.	Silica	rr651	T. Nash et al. 9950 (NY)	USA	Arizona	OQ190676	OQ183692		OQ151766	OQ116514
Ditaxis lanceolata (Benth.) Pax & K.Hoffm.	Genbank	GBBoyd10488	Boyd 10488 (CA)	USA		MF979102.1				
Ditaxis lanceolata (Benth.) Pax & K.Hoffm.	Genbank	GBSanders3529	A. C. Sanders et al. 3529 (MEXU)	Mexico				HG97203.2	HG97186.5	
Ditaxis lanceolata (Benth.) Pax & K.Hoffm.	Herbarium	YPR0	Y. Ramírez-Amezcua, V.W. Steinmann & Y. Yang, 1506 (IEB)	Mexico	Baja California Sur. Municipio de La Paz	OQ190675				
Ditaxis malpighiacea (Ule) Pax & Hoffm.	Silica	rr652	J. Külkamp et al. 659 (RB)	Brasil	Bahía, Mucugê	OQ190677	OQ183693		OQ151767	OQ116515
Ditaxis malpighiacea (Ule) Pax & Hoffm.	Silica	rr653	J. Külkamp et al. 692 (RB)	Brasil	Bahía, Morro do Chapéu	OQ190678	OQ183694		OQ151768	

Ditaxis malpighiphila (Hicken) L.C.Wheeler	Silica	rr654	J. Külkamp 1245 (BA)	Argentina	Mendoza, Luján de Cuyo, Potrerillos	OQ190679	OQ18369 5		OQ151769	OQ116513
Ditaxis manzanilloana (Rose) Pax & K.Hoffm.	Herbarium	y133	R. Guerrero & Van Devender 2009-1003 (IEB)	México	Sonora	OQ190681	OQ18369 6			OQ116501
Ditaxis manzanilloana (Rose) Pax & K.Hoffm.	Genbank	GBSaynes5417	A. Saynes et al. (5417 MEXU)	Mexico				HG97203.3	HG97186.6	
Ditaxis manzanilloana (Rose) Pax & K.Hoffm.	Herbarium	SonoraYPR	R. Guerrero & Van Devender 2009-1003 (1) (IEB)	México	Sonora	OQ190680				
Ditaxis mercurialina (Nutt.) J.M.Coult.	Herbarium	YPRTR	C.A. Morse 6830 (NY)	USA	Kansas, Chase	OQ190684			OQ151772	
Ditaxis mercurialina (Nutt.) J.M.Coult.	Herbarium	rr655	C.A. Morse 6830 (1) (NY)	USA	Kansas, Chase Co: N side of Chase Co State Fishing Lake	OQ190682			OQ151770	OQ116499
Ditaxis mercurialina var. *pilosissima* (Bentham) A. Heller	Herbarium	rr656	W.R. Carr 32893 (NY)	USA	Texas	OQ190683	OQ18369 7		OQ151771	OQ116500
Ditaxis montevidensis (Didr.) Pax	Herbarium	y98	T. Meyer 2225 (F)	Argentina	Fontana	OQ190651	OQ18367 9		OQ151748	
Ditaxis montevidensis (Didr.) Pax	Herbarium	YPR	T. Meyer 2225 (1) (F)	Argentina	Fontana	OQ190652				
Ditaxis montevidensis (Didr.) Pax	Silica	rr657	J. Külkamp et al. 367 (ICN)	Brasil	Río Grande do Sul, Barra do Quaraí	OQ190685	OQ18369 8		OQ151774	
Ditaxis montevidensis (Didr.) Pax	Silica	rr658	J. Külkamp 1250 (BA)	Argentina	Córdoba, Córdoba capital	OQ190686	OQ18369 9		OQ151775	OQ116511

Ditaxis montevidensis (Didr.) Pax	Herbarium	rr677	R. Riina 1892 (MA)	Argentina	Salta	OQ190687	OQ18370 0		OQ151776	OQ116510
Ditaxis montevidensis (Didr.) Pax	Herbarium	rr667	A. Pott 8878 (CPAP)	Brasil	Mato Grosso do Sul, Porto Murtinho	OQ190706			OQ151798	
Ditaxis montevidensis (Didr.) Pax	Herbarium	YPR1	Wood & Carretero 19309 (LPB)	Bolivia	Dpto. Chuquisaca. Prov. Oropeza				OQ151777	
Ditaxis polygama (Jacq.) L.C.Wheeler	Herbarium	rr659	R.A. Howard 18661 (NY)	Francia (West Indies)	Martinique, Sainte-Anne, Creve-Coeur	OQ190689			OQ151779	
Ditaxis polygama (Jacq.) L.C.Wheeler	Herbarium	YPR	R.A. Howard 18661 (1) (NY)	Francia (West Indies)	Martinique, Sainte-Anne, Creve-Coeur		OQ18370 1		OQ151780	
Ditaxis pringlei (Greenm.) Pax & K.Hoffm.	Silica	rr660	Y. Ramirez-Amezcua 513 (NY)	Mexico	Michoacán, Uruapan	OQ190690	OQ18370 2		OQ151781	OQ116516
Ditaxis pringlei (Greenm.) Pax & K.Hoffm.	Silica	YPR	R. Ramírez-Amezcua, A.G. Rocha-Loredo & A. Machuca 656 (IEB)	Mexico	Jalisco. Mpio. Tuxcueca	OQ190691			OQ151782	
Ditaxis pringlei (Greenm.) Pax & K.Hoffm.	Genbank	GBRivera3326	J. Rivera & Espinosa 3326 (MEXU)	Mexico				HG97203.5	HG97186.8	
Ditaxis purpurascens (S.Moore) Pax & K.Hoffm.	Silica	rr661	J. Külkamp 513 (RB)	Brasil	Mato Grosso do Sul, Corumbá	OQ190692	OQ18370 3		OQ151783	OQ116517
Ditaxis purpurascens (S.Moore) Pax & K.Hoffm.	Herbarium	rr678	S. Beck 27535 (MA)	Bolivia	Santa Cruz	OQ190693	OQ18370 4		OQ151784	
Ditaxis rubricaulis Pax & K.Hoffm.	Herbarium	y91	Croizat s.n. (NY)	Venezuel a	Sucre	OQ190694			OQ151786	
Ditaxis salina Pax & K.Hoffm.	Silica	rr662	A. Pott et al. 10785 (CPAP)	Brasil	Mato Grosso do Sul, Bela Vista	OQ190695	OQ18370 5		OQ151787	

Ditaxis sellowiana Pax & K.Hoffm.	Herbarium	YPR1	Jorgensen 4352 (F)	Paraguay	Villarrica				OQ151785	
Ditaxis sellowiana Pax & K.Hoffm.	Silica	rr663	J. Külkamp et al. 418 (ICN)	Brasil	Río Grande do Sul, Santiago	OQ190696	OQ18370 6		OQ151789	
Ditaxis sellowiana Pax & K.Hoffm.	Silica	rr664	J. Külkamp et al. 394 (ICN)	Brasil	Río Grande do Sul, Alegrete	OQ190694	OQ18370 7		OQ151790	OQ116518
Ditaxis sellowiana Pax & K.Hoffm.	Külkamp-Masters	K9	J. Külkamp et al. 159 (ICN)	Brasil	Rio Grande do Sul, Caçapava do Sul				OQ151788	
Ditaxis sellowiana Pax & K.Hoffm.	Herbarium	y84	L.T.M. Pedersen 3807 (NY)	Argentina		OQ190637	OQ18366 8			
Ditaxis sellowiana Pax & K.Hoffm.	Herbarium	YPR	L.T.M. Pedersen 3807 (1) (NY)	Argentina					OQ151732	
Ditaxis sellowiana Pax & K.Hoffm.	Herbarium	y87	Jorgensen 351 (F)	Argentina		OQ190698			OQ151791	
Ditaxis serrata Torr.	Herbarium	YPR1	T. Van Devender & Reina 2007-09 (IEB)	Mexico	Sonora. Arroyo El Mentidero	OQ190688			OQ151778	
Ditaxis serrata Torr.	Silica	rr665	V.W. Steinmann 1709 (NY)	Mexico	Michoacán, Uruapan	OQ190699	OQ18370 8		OQ151792	OQ116519
Ditaxis serrata Torr.	Herbarium	y101	Y. Ramírez-Amezcua et al. 1068 (IEB)	Mexico	Michoacán				OQ151773 OQ151793	OQ116520
Ditaxis silviae = *Argythamnia silviae* Ram.-Amezcua & V.W. Steinm.	Herbarium	rr668	S. Salas et al. 3496 (MEXU)	Mexico	Oaxaca, Tehuantepec, San Pedro Huamelula	OQ190707	OQ18371 0		OQ151799	
Ditaxis silviae = *Argythamnia silvia* Ram.-Amezcua & V.W. Steinm.	Herbarium	YPR	S. Salas et al. 3496 (1) (MEXU)	Mexico	Oaxaca, Tehuantepec, San Pedro Huamelula	OQ190702				

Ditaxis silviae = *Argythamnia silvia* Ram.-Amezcua & V.W. Steinm.	Herbarium	y97	S. Salas et al. 3496 (2) (MEXU)	Mexico	Oaxaca, Tehuantepec, San Pedro Huamelula,	OQ190701			OQ151802	
Ditaxis simoniana Casar.	Silica	rr666	J. Külkamp 482 (RB)	Brasil	Rio de Janeiro, Niterói	OQ190704	OQ183709		OQ151795	
Ditaxis simulans (J.W.Ingram) Radcl.-Sm. & Govaerts	Herbarium	YPR	D.H. Riskind 2370 (MEXU)	Mexico	Coahuila. Mpio. Villa Acuña	OQ190705			OQ151797	
Ditaxis sp.	Silica	rr633	J. Carrion 2005 (HUEFS)	Brasil	Minas Gerais, Januária	OQ190640	OQ183674		OQ151736	OQ116504
Ditaxis sp.	Silica	rr634	J. Külkamp et al. 914 (RB)	Brasil	Minas Gerais, Santo Hipólito	OQ190641	OQ183675		OQ151737	OQ116503
Ditaxis sp.	Herbarium	rr632	W.W. Thomas, J. Jardim & F. Juchum 12361 (NY)	Brasil	Bahía, Itaju do Colônia	OQ190703	OQ183673		OQ151794	OQ116506
Ditaxis sp.	Herbarium	y95	W.W. Thomas, J. Jardim & F. Juchum 12361 (1) (NY)	Brasil	Bahía, Itaju do Colônia	OQ190660				
Ditaxis sp.	Herbarium	YPRItaju	W.W. Thomas, J. Jardim & F. Juchum 12361 (2) (NY)	Brasil	Bahía, Itaju do Colônia	OQ190661			OQ151796	
Enriquebeltrania crenatifolia (Miranda) Rzed.	Genbank	Denova114	De-Nova 114 (XAL)	Mexico		DQ997793.1				
Enriquebeltrania disjuncta De-Nova & Sosa	Genbank	DeNova109	A. De Nova 109 (XAL)	Mexico		DQ997794.1				
Enriquebeltrania disjuncta De-Nova & Sosa	Genbank	DeNova107	A. De Nova 107 (MEXU)	Mexico				HG972036.1	HG971869.1	
Philyra brasiliensis Klotzsch	Silica	rr671	J. Külkamp et al. 1046 (RB)	Brasil	Espírito Santo, Colatina	OQ190710	OQ183711		OQ151803	
Philyra brasiliensis Klotzsch	Silica	rr672	J. Külkamp et al. 1213 (RB)	Brasil	Río de Janeiro, Italva	OQ190711	OQ183712		OQ151804	OQ116533

Philyra brasiliensis Klotzsch	Genbank	GBZardini	E. Zardini s.n. (K)	Paraguay				HG97208.1	HG97191.4	
Philyra brasiliensis Klotzsch	Genbank	YPR	G. Webster 25536 (NY)	Brazil		MK780962.1	MK7811 11.1		AY794740.1	
Philyra brasiliensis Klotzsch	Genbank	GBThomas12565	W.W. Thomas et al. 12565 (NY)	Brazil		GU000028.1				
Plukenetia penninervia Müll.Arg.	Genbank	Frisch5996	Wallnoefer & Frisch 5996 (MO)	Guatemal a		MF502551.1	MF50247 7.1			
Plukenetia penninervia Müll.Arg.	Genbank	Calonico22458	J. Calónico 22458 (MEXU)	Mexico					HG971924.1	
Plukenetia volubilis L.	Genbank	Huamantupa3500	Huamantupa 3500 (CAN)	Peru		MF502500.1	MF50256 8.1			
Plukenetia volubilis L.	Genbank	Armbruster38	Armbruster 38 (no collection cited)	No country cited					AY794756.1	
Seidelia triandra (E. Mey.) Pax	Genbank	Giess13381	Giess 13381 (MO)	No country cited					AY794762.1	
