# Supplementary material for: Systematics of Ditaxinae and Related Lineages within the Subfamily Acalyphoideae (Euphorbiaceae) Based on Molecular Phylogenetics
Source: Biology (Basel). 2023 Jan 21;12(2):173. doi: 10.3390/biology12020173 (PMC9952443; doi:10.3390/biology12020173)

SUPPLEMENTARY MATERIAL S2

Systematics of Ditaxinae and related lineages within subfamily Acalyphoideae (Euphorbiaceae) based on molecular phylogenetics

Josimar Külkamp, Ricarda Riina, Yocupitzia Ramírez-Amezcua, João R.V. Iganci, Inês Cordeiro, Raquel González-Páramo, Sabina Irene Lara-Cabrera and José Fernando A. Baumgratz

Supplementary material S2: Supplementary tables and figures.

TABLE S1: Extraction steps by the CTAB method adapted from Doyle (1991)

Steps	Proceedings
1	Buffer CTAB/sample = 0.02 ml CTAB (2% p/v); 0.02 ml PVP [Pm40,000] (2% p/v); 0.28 ml NaCl (1.4 M); 0.04 ml EDTA pH 8 (20 mM); 0.1 ml Tris-HCL pH 8 (100 mM); 0,02 ml 2-mercaptoetanol (2% v/v); 0.56 ml H2O. Obs: Add the solid components over the liquids and bake at 65 °C for 1 hr.
2	Macerate 15 mg of dehydrated tissue in TissueLyser II (Quiagen) for 10 min.
3	Add 950 ul of the CTAB Buffer to each tube of already macerated sample and homogenize.
4	Place the tubes in a thermoblock for 1 h at 65°C with constant agitation at 650-700 rpm.
5	Add 700 µl of chloroform (isoamyl NO) to each tube
6	Centrifuge the tubes for 15 min at 13,000 rpm at 4°C
7	Transfer the supernatant (ca. 800 ul) to a new tube
8	Add 550 µl of isopropanol (stored in the freezer) to each tube and homogenize.
9	Store in the freezer -20°C for 24-48 hours
10	Centrifuge the tubes for 15 min at 13,000 rpm at 4°C
11	Discard all the supernatant, leaving only the pellet adhering to the bottom of the tube.
12	Add 500 ul of 70% ethanol to each tube and vortex to loosen the pellet from the bottom of the tube.
13	Repeat step 10
14	Repeat step 11
15	Repeat step 12
16	Repeat step 10
17	Discard the liquid and place the open tubes at 37 °C for 1 hr to dry all the liquid.
18	Add 50 ul MilliQ water at 65°C and store at 4°C for use or dilution.

Reference

Doyle, J. DNA Protocols for Plants. In: Hewitt, G.M.; Johnston, A.W.B.; Young, J.P.W., Eds., Molecular Techniques in Taxonomy, Springer, Berlin, Heidelberg, **1991**. 283-293.

TABLE S2. List of molecular markers, primer sequence and references

Region	Primer name	Primer sequence (5′ to 3′)	Reference
ETS	ETS_F2	ATGATCGTTTGSTTTGGCAGGCTC	Wurdack, K. *Com. Pers.*
	18S_5R	GAATTAGTTCATACTTACACATGCATG	Wurdack, K. *Com. Pers.*
	18S_5prR	CTGGCAGGATCAACCAGGTAGCA	Cardinal-McTeague *et al.* (2019)
	ETS_F3F	GTGTCGTGCTCTCGGATGC	Cardinal-McTeague *et al.* (2019)
*pet*B-D	pipetB1411F	GCCGTMTTTATGTTAATGC	Löhne & Borsch, 2005
	pipetD738R	AATTTAGCYCTTAATACAGG	Löhne & Borsch, 2005
	EPHpetD657F	TTTATRATACTATCGGAGTG	Cervantes *et al.* 2016
	EPHpetD891R	TAAATGCTCAATRCCCAAG	Cervantes *et al.* 2016
ITS	ITSw1f	CCTTATCATTTAGAGGAAGGAG	Silva *et al.* 2020
	ITSw2r	TATGCTTAAAYTCAGCGGGT	Silva *et al.* 2020
	ITSp2r	GCCRAGATATCCGTTGCCGAG	Cheng *et al.* 2016
	ITSp3f	YGACTCTCGGCAACGGATA	Cheng *et al.* 2016
*trn*L-F	trnTc	CGAAATCGGTAGACGCTACG	Taberlet *et al.* 1991
	trnTd	GGGGATAGAGGGACTTGAAC	Taberlet *et al.* 1991
	trnTe	GGTTCAAGTCCCTCTATCCC	Taberlet *et al.* 1991
	trnTf	ATTTGAACTGGTGACACGAG	Taberlet *et al.* 1991
trnT-L	trnA2	CAAATGCGATGCTCTAACCT	Shaw *et al.* 2007
	trnB	TCTACCGATTTCGCCATATC	Shaw *et al.* 2007

References

Cardinal-McTeague, W.M.; Wurdack, K.J.; Sigel, E.M.; Gillespie, L.J. Seed size evolution and biogeography of *Plukenetia* (Euphorbiaceae), a pantropical genus with traditionally cultivated oilseed species. *BMC Evolutionary Biology* **2019**, 19: 29, Doi: 10.1186/s12862-018-1308-9

Cervantes, A.; Fuentes, S.; Gutiérrez, J.; Magallón. S.; Borsch, T. Successive arrivals since the Miocene shaped the diversity of the Caribbean Acalyphoideae (Euphorbiaceae). *Journal of Biogeography* **2016**, 43, 1773–1785, Doi: 10.1111/jbi.12790.

Cheng, T.; Xu, C.; Lei, L.; Li, C.; Zhang, Y.; Zhou, S. Barcoding the kingdom Plantae: new PCR primers for ITS regions of plants with improved universality and specificity. *Molecular Ecology Resources* **2016**, 16: 138–149, Doi: 10.1111/1755-0998.12438.

Löhne, C.; Borsch, T. Molecular Evolution and Phylogenetic Utility of the *pet*D Group II Intron: A Case Study in Basal Angiosperms. *Molecular Biology and Evolution* **2005**, 22: 317–332. Doi: 10.1093/molbev/msi019.

Shaw, J.; Lickey, E.B.; Schilling, E.E.; Small, R.L. Comparison of whole chloroplast genome sequences to choose noncoding regions for phylogenetic studies in angiosperms: the tortoise and the hare III. *American Journal of Botany* **2007**, 94: 275–288.

Silva, O.L.M.; Riina, R.; Cordeiro, I. Phylogeny and biogeography of *Astraea* with new insights into the evolutionary history of Crotoneae (Euphorbiaceae). *Molecular Phylogenetics and Evolution* **2020**, 145: 16, Doi: 10.1016/j.ympev.2020.106738.

Taberlet, P.; Gielly, L.; Pautou, G.; Bouvet, J. Universal primers for amplification of three non-coding regions of chloroplast DNA. *Plant Molecular Biology* **1991**, 17: 1105-1109.

PCRs support information

The PCR amplifications were conducted in thermocyclers Eppendorf Mastercycler X50s PC, with 25uL reactions (thermocycler temperature protocols see Supplementary materials S2: Table S3 [below]). Each reaction tube includes 12uL of MyTaq Red Mix (Bioline); 10uL H2O; 1uL 10uM of each primer and 1uL Genomic DNA. For samples of difficult amplification, PuReTaq Ready-To-Go PCR Beads (GE Healthcare) were used, added up 22uL H2O; 1uL 10uM of each primer and 1uL Genomic DNA. All prc products were analyzed on agarose gel with 4 uL per well (1 blue dye + 3 uL PCR product), if reactions used MyTaq Red Mix, use 4uL PCR product, later the gel was checked under UV light. The PCR reactions that presented amplified DNA, were purified with ExoSap PCR Purification, 6,5 uL (1:10 concentration) was added to each tube. Afterwards, the tubes were submitted to a program 15 min at 37 C + 15 min 80 C in the thermocycler. For sequencing plates, 5 µl purified DNA + 5 µl 5mM primers was added to each sample and sent for sequencing at MACROGEN (Macrogen, Madrid, Spain), using the same amplification primers (Supplementary materials S2: Table 2).

TABLE S3. Amplification and sequencing protocols

Region	Amplification primers		Thermocycler protocols
	Dry samples on silica gel	Herbarium samples	
ETS	ETS_F2 + 18S_5R	(ETS_F2 + 18S_5prR) and (ETS_F3F + 18S_5R)	Initial denaturation step = 5 min at 95 °C<br>34 cycles of denaturation = 1 min at 95 °C<br>Annealing = 1 min at 52 °C<br>Elongation = 1 min at 72°C<br>Final extension = 10 min at 72 °C
ITS	ITSw1f + ITSw2r	(ITSw1f + ITSp2r) and (ITSp3f + ITSw2r)	Initial denaturation step = 5 min at 95 °C<br>36 cycles of denaturation = 1 min at 95 °C<br>Annealing = 1.5 min at 50 °C<br>Elongation = 45 sec at 72°C<br>Final extension = 10 min at 72 °C
*pet*B-D	pipetB1411F + pipetD738R	(pipetB1411F + EPHpetD891R) and (EPHpetD657F + pipetD738R)	Initial denaturation step = 5 min at 94 °C<br>30 cycles of denaturation = 1 min at 94 °C<br>Annealing = 1 min at 50 °C<br>Elongation = 2 min at 72°C<br>Final extension = 15 min at 72 °C
*trn*L-F	trnTc + trnTf	(trnTc + trnTd) and (trnTe + trnTf)	Initial denaturation step = 2 min at 94 °C<br>36 cycles of denaturation = 1 min at 95 °C<br>Annealing = 2 min at 53 °C<br>Elongation = 1 min at 72°C<br>Final extension = 10 min at 72 °C
*trn*T-L	trnA2+trnB	trnA2+trnB	Initial denaturation step = 5 min at 95 °C<br>30 cycles of denaturation = 30 sec at 95 °C<br>Annealing = 1 min at 52 °C<br>Elongation = 1 min at 72°C<br>Final extension = 10 min at 72 °C

Figures S1-S9

The phylogenetic trees of the individual markers, the concatenated matrices of plastid and nuclear markers are presented below.

Figure S1: Consensus tree of the *trn*T marker obtained by Bayesian Inference performed in MrBayes. Numbers indicate Bayesian posterior probabilities

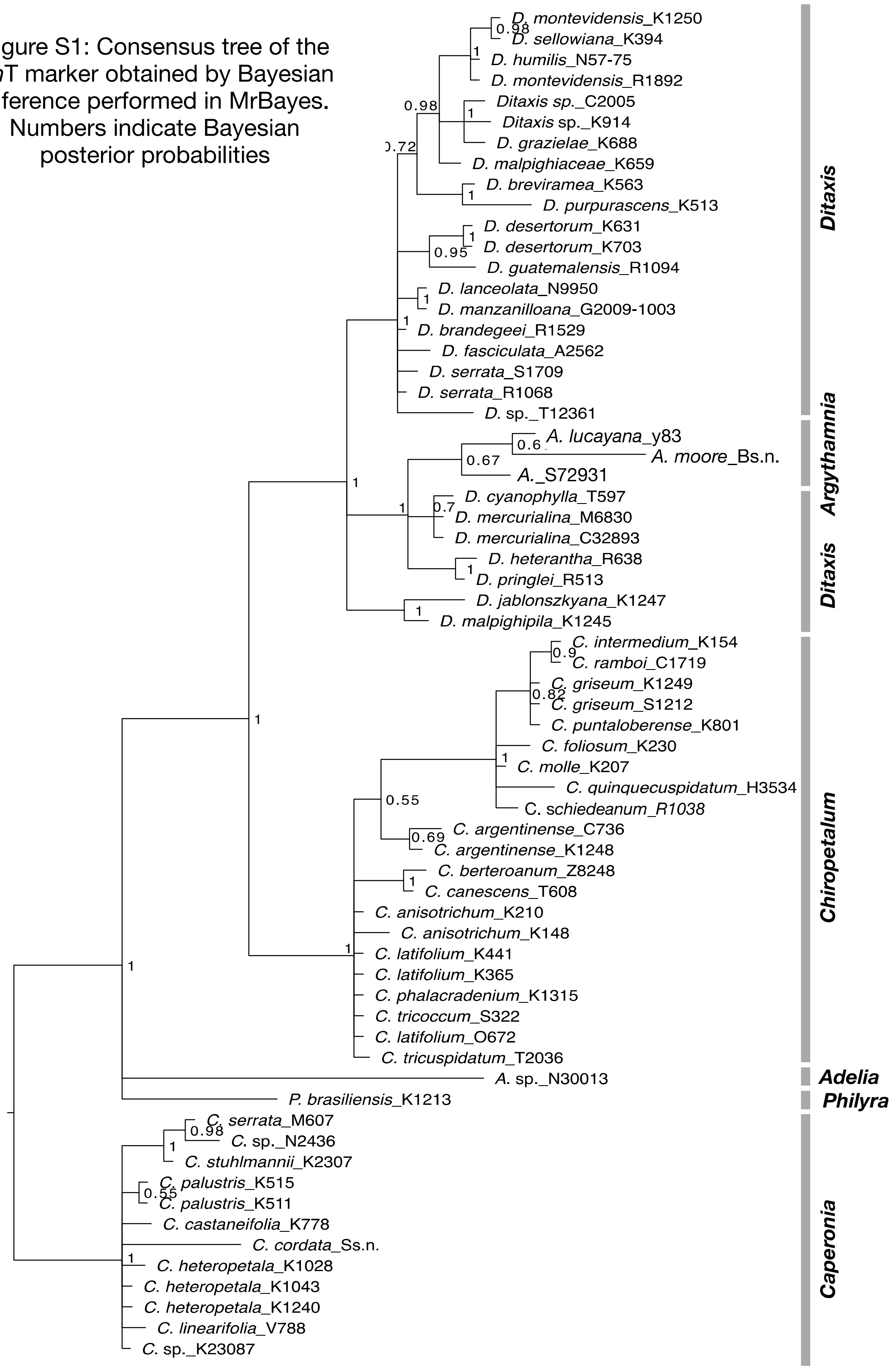

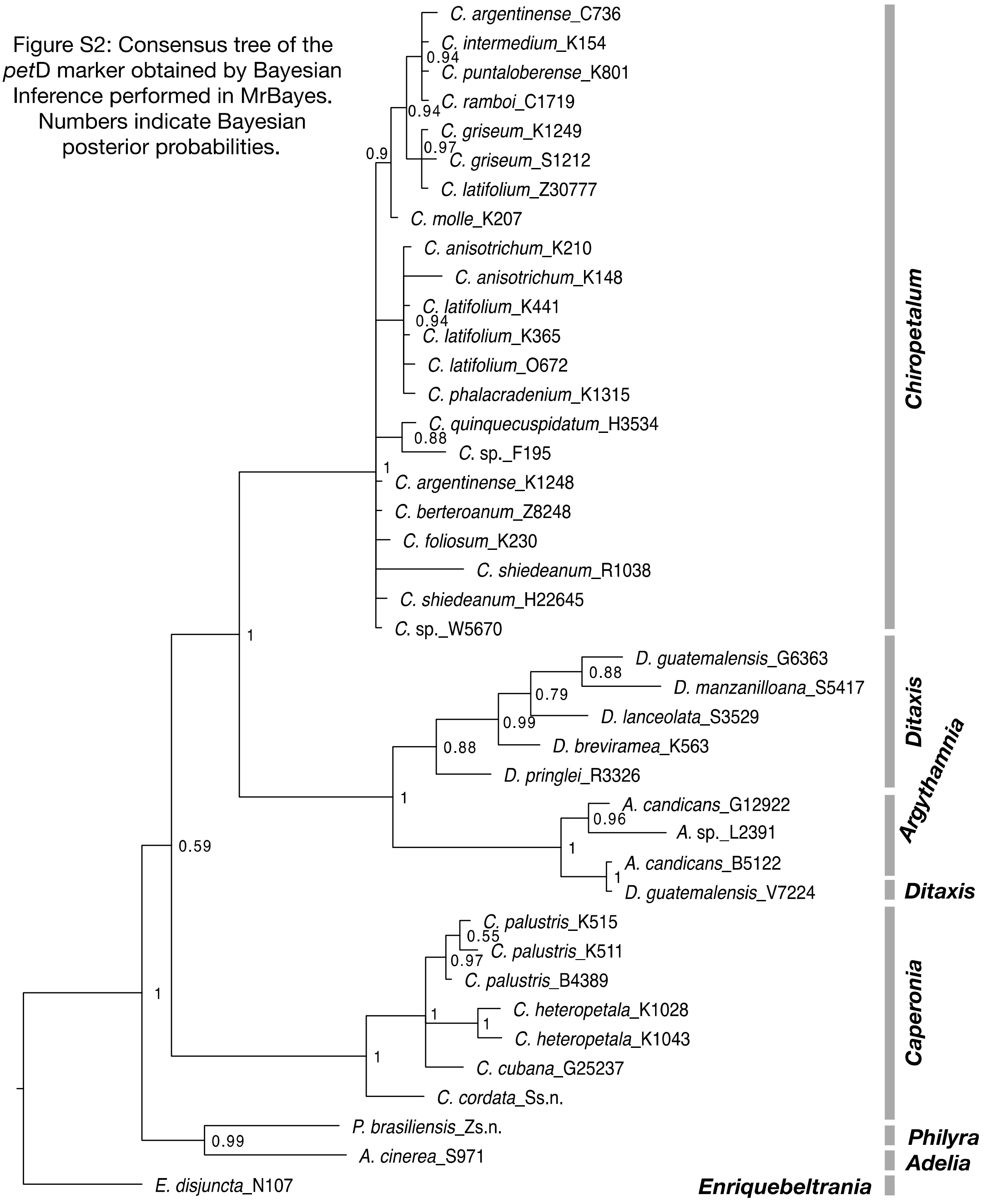

Figure S2: Consensus tree of the *pet*D marker obtained by Bayesian Inference performed in MrBayes. Numbers indicate Bayesian posterior probabilities.

Figure S3: Consensus tree of the *trn*L-F marker obtained by Bayesian Inference performed in MrBayes. Numbers indicate Bayesian posterior probabilities.

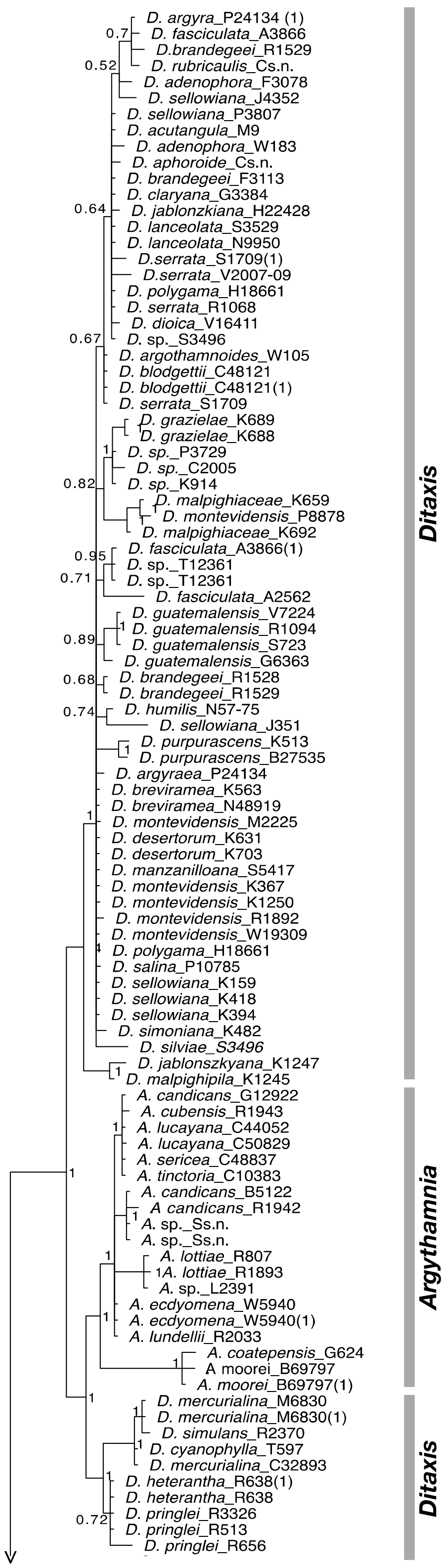

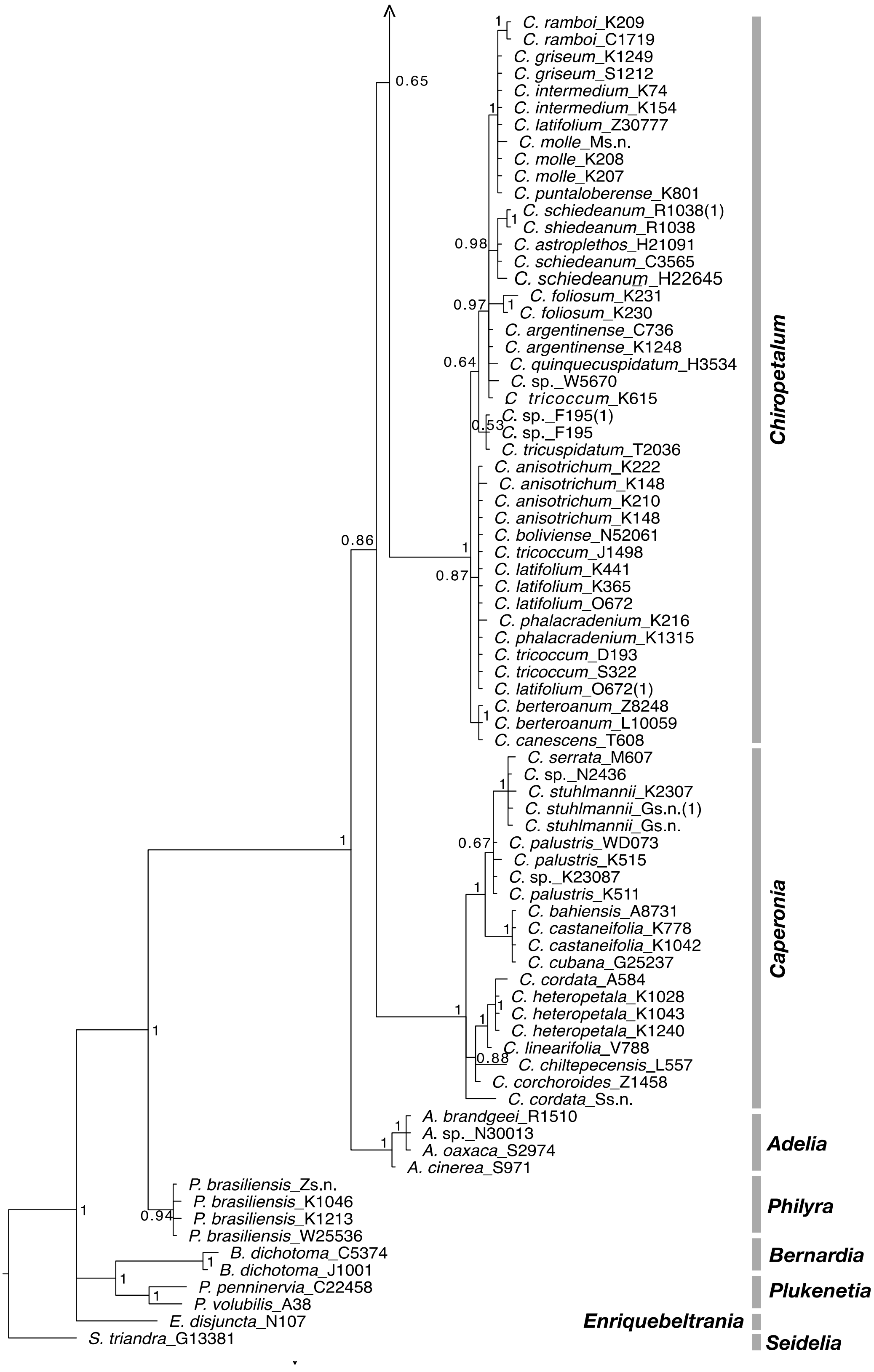
C. ramboi_K209
C. ramboi_C1719
C. griseum_K1249
C. griseum_S1212
C. intermedium_K74
C. intermedium_K154
C. latifolium_Z30777
C. molle_Ms.n.
C. molle_K208
C. molle_K207
C. puntaloberense_K801
C. schiedeanum_R1038(1)
C. shiedeanum_R1038
C. astroplethos_H21091
C. schiedeanum_C3565
C. schiedeanum_H22645
C. foliosum_K231
C. foliosum_K230
C. argentinense_C736
C. argentinense_K1248
C. quinquecuspidatum_H3534
C. sp._W5670
C tricoccum_K615
C. sp._F195(1)
C. sp._F195
C. tricuspidatum_T2036
C. anisotrichum_K222
C. anisotrichum_K148
C. anisotrichum_K210
C. anisotrichum_K148
C. boliviense_N52061
C. tricoccum_J1498
C. latifolium_K441
C. latifolium_K365
C. latifolium_O672
C. phalacradenium_K216
C. phalacradenium_K1315
C. tricoccum_D193
C. tricoccum_S322
C. latifolium_O672(1)
C. berteroanum_Z8248
C. berteroanum_L10059
C. canescens_T608
C. serrata_M607
C. sp._N2436
C. stuhlmannii_K2307
C. stuhlmannii_Gs.n.(1)
C. stuhlmannii_Gs.n.
C. palustris_WD073
C. palustris_K515
C. sp._K23087
C. palustris_K511
C. bahiensis_A8731
C. castaneifolia_K778
C. castaneifolia_K1042
C. cubana_G25237
C. cordata_A584
C. heteropetala_K1028
C. heteropetala_K1043
C. heteropetala_K1240
C. linearifolia_V788
C. chiltepecensis_L557
C. corchoroides_Z1458
C. cordata_Ss.n.
A. brandgeei_R1510
A. sp._N30013
A. oaxaca_S2974
A. cinerea_S971
P. brasiliensis_Zs.n.
P. brasiliensis_K1046
P. brasiliensis_K1213
P. brasiliensis_W25536
B. dichotoma_C5374
B. dichotoma_J1001
P. penninervia_C22458
P. volubilis_A38
E. disjuncta_N107
S. triandra_G13381
0.65
0.86
1
0.98
0.97
0.64
0.53
0.87
0.67
0.88
0.94
Chiropetalum
Caperonia
Adelia
Philyra
Bernardia
Plukenetia
Enriquebeltrania
Seidelia

Figure S4: Consensus tree of the ITS marker obtained by Bayesian Inference performed in MrBayes. Numbers indicate Bayesian posterior probabilities.

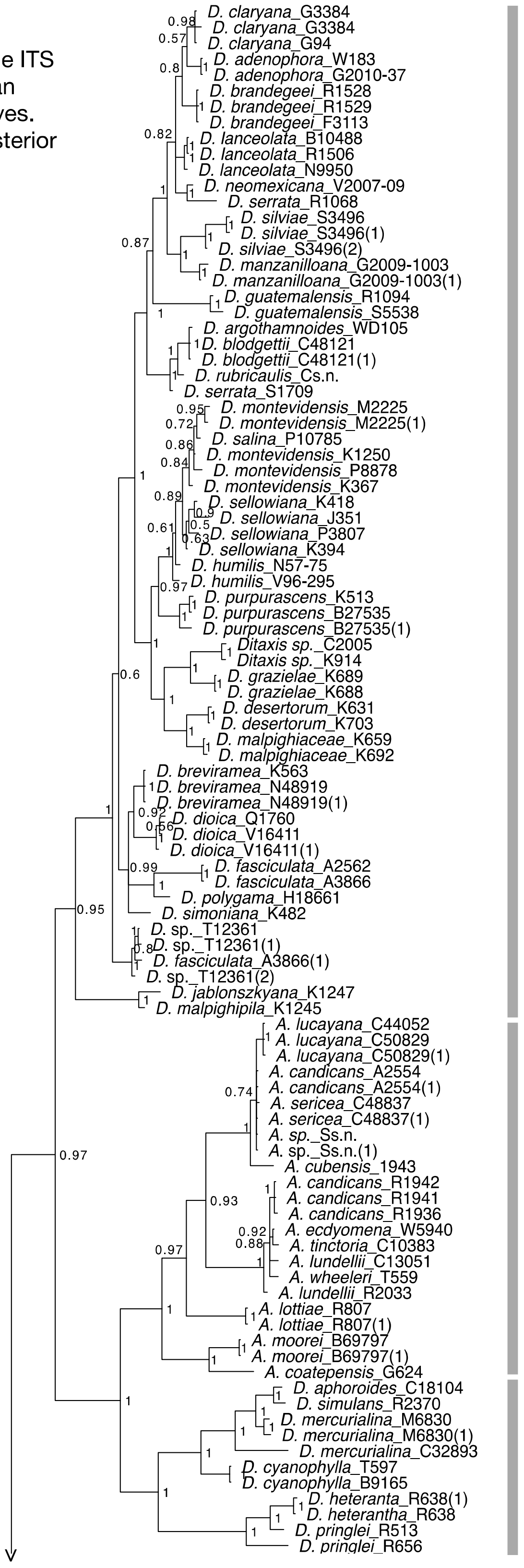

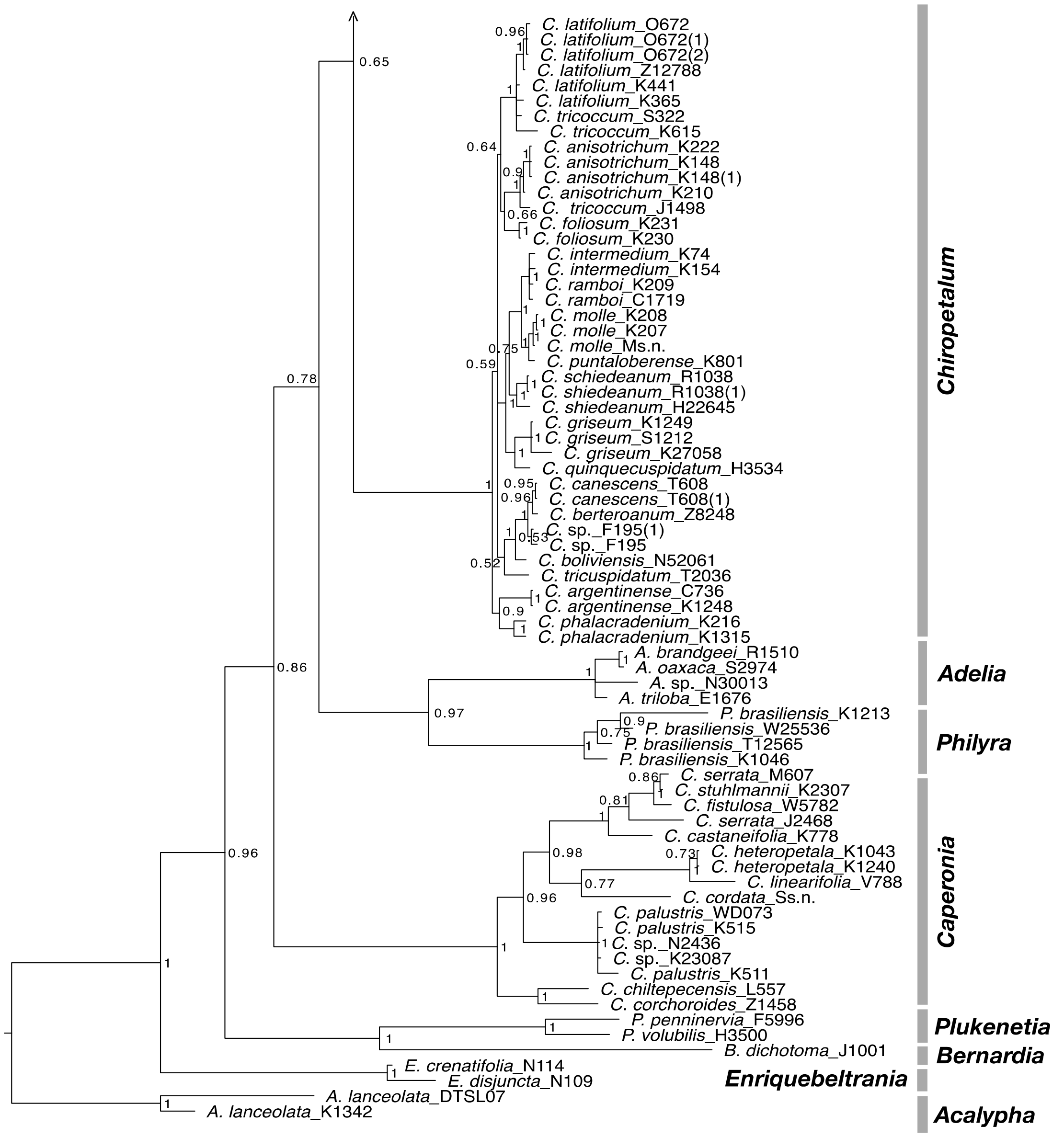
C. latifolium_O672
C. latifolium_O672(1)
C. latifolium_O672(2)
C. latifolium_Z12788
C. latifolium_K441
C. latifolium_K365
C. tricoccum_S322
C. tricoccum_K615
C. anisotrichum_K222
C. anisotrichum_K148
C. anisotrichum_K148(1)
C. anisotrichum_K210
C. tricoccum_J1498
C. foliosum_K231
C. foliosum_K230
C. intermedium_K74
C. intermedium_K154
C. ramboi_K209
C. ramboi_C1719
C. molle_K208
C. molle_K207
C. molle_Ms.n.
C. puntaloberense_K801
C. schiedeanum_R1038
C. shiedeanum_R1038(1)
C. shiedeanum_H22645
C. griseum_K1249
C. griseum_S1212
C. griseum_K27058
C. quinquecuspidatum_H3534
C. canescens_T608
C. canescens_T608(1)
C. berteroanum_Z8248
C. sp._F195(1)
C. sp._F195
C. boliviensis_N52061
C. tricuspidatum_T2036
C. argentinense_C736
C. argentinense_K1248
C. phalacradenium_K216
C. phalacradenium_K1315
A. brandgeei_R1510
A. oaxaca_S2974
A. sp._N30013
A. triloba_E1676
P. brasiliensis_K1213
P. brasiliensis_W25536
P. brasiliensis_T12565
P. brasiliensis_K1046
C. serrata_M607
C. stuhlmannii_K2307
C. fistulosa_W5782
C. serrata_J2468
C. castaneifolia_K778
C. heteropetala_K1043
C. heteropetala_K1240
C. linearifolia_V788
C. cordata_Ss.n.
C. palustris_WD073
C. palustris_K515
C. sp._N2436
C. sp._K23087
C. palustris_K511
C. chiltepecensis_L557
C. corchoroides_Z1458
P. penninervia_F5996
P. volubilis_H3500
B. dichotoma_J1001
E. crenatifolia_N114
E. disjuncta_N109
A. lanceolata_DTSL07
A. lanceolata_K1342
Chiropetalum
Adelia
Philyra
Caperonia
Plukenetia
Bernardia
Enriquebeltrania
Acalypha

Figure S5: Consensus tree of the ETS marker obtained by Bayesian Inference performed in MrBayes. Numbers indicate Bayesian posterior probabilities.

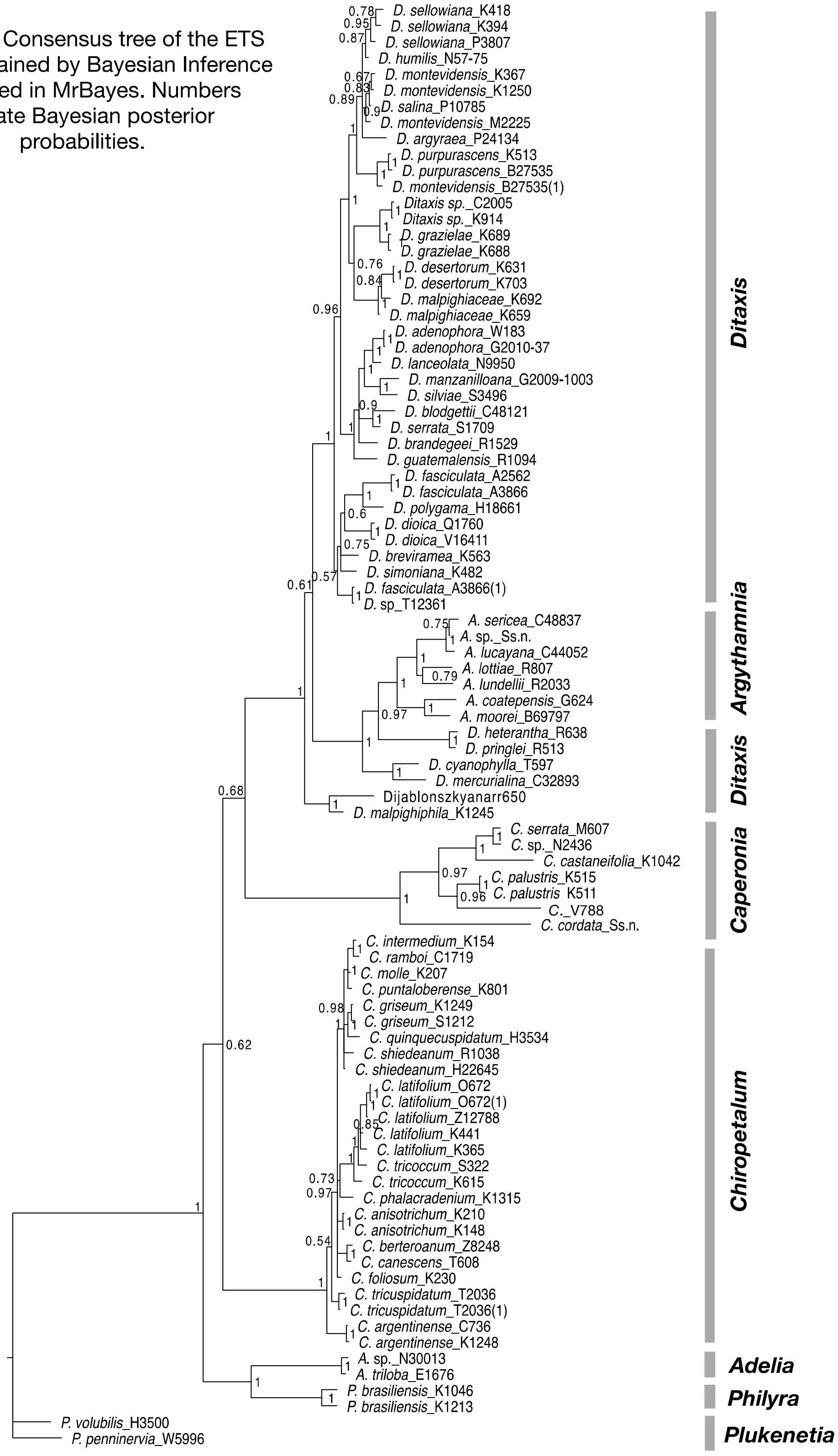

Figure S6: Consensus tree of the plastid markers (*trn*L-F+*trn*T+*pet*D) obtained by Bayesian Inference performed in MrBayes. Numbers indicate Bayesian posterior probabilities.

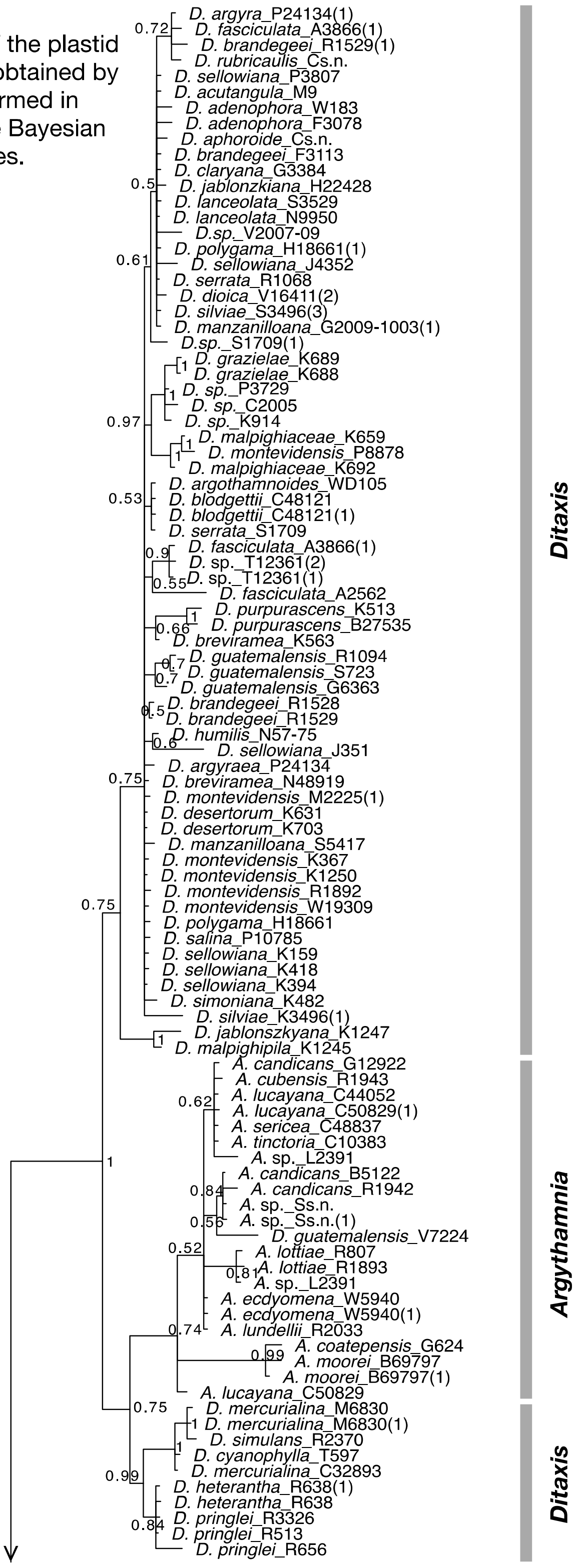

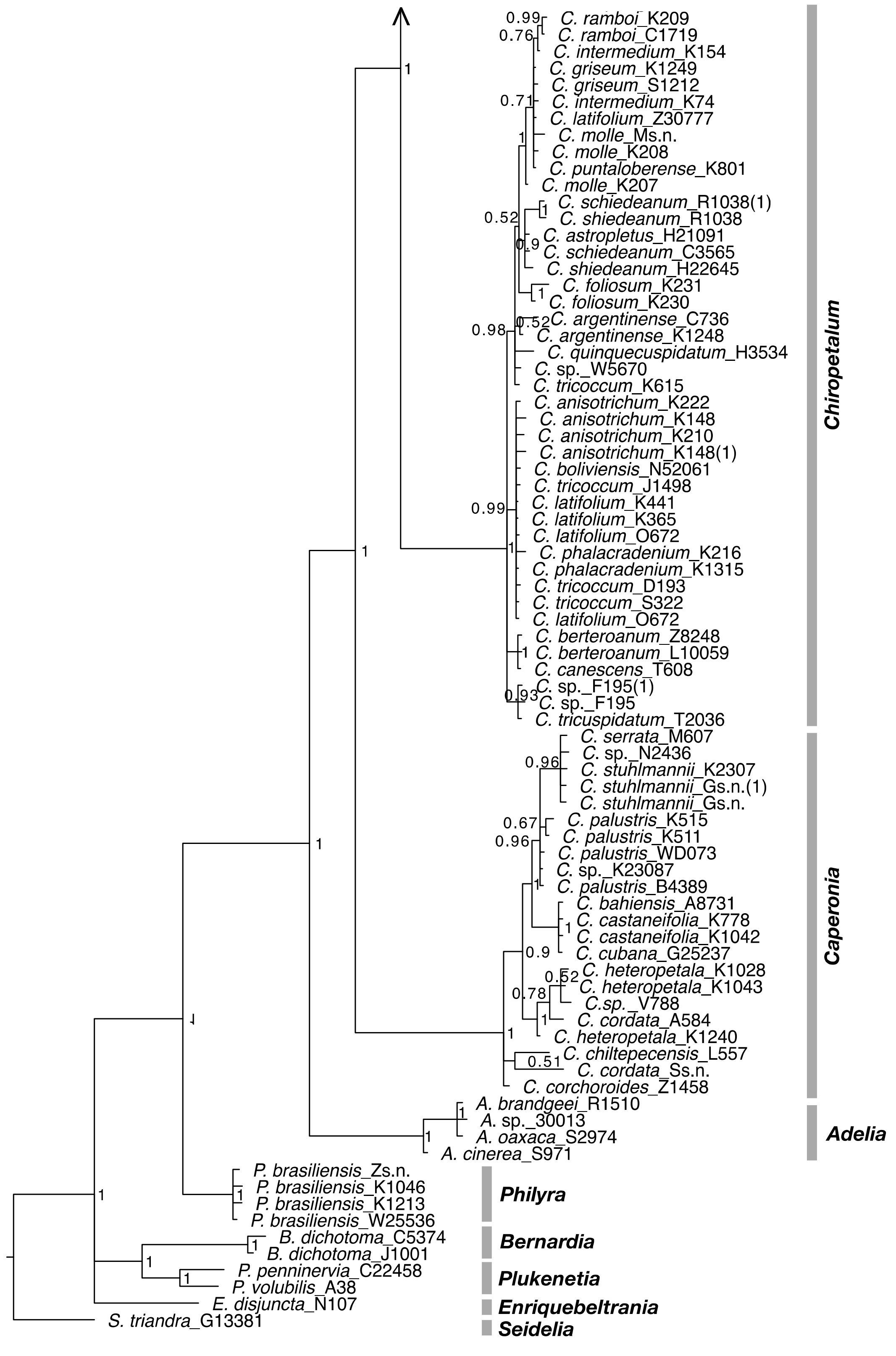

C. ramboi_K209
C. ramboi_C1719
C. intermedium_K154
C. griseum_K1249
C. griseum_S1212
C. intermedium_K74
C. latifolium_Z30777
C. molle_Ms.n.
C. molle_K208
C. puntaloberense_K801
C. molle_K207
C. schiedeanum_R1038(1)
C. shiedeanum_R1038
C. astropletus_H21091
C. schiedeanum_C3565
C. shiedeanum_H22645
C. foliosum_K231
C. foliosum_K230
C. argentinense_C736
C. argentinense_K1248
C. quinquecuspidatum_H3534
C. sp._W5670
C. tricoccum_K615
C. anisotrichum_K222
C. anisotrichum_K148
C. anisotrichum_K210
C. anisotrichum_K148(1)
C. boliviensis_N52061
C. tricoccum_J1498
C. latifolium_K441
C. latifolium_K365
C. latifolium_O672
C. phalacradenium_K216
C. phalacradenium_K1315
C. tricoccum_D193
C. tricoccum_S322
C. latifolium_O672
C. berteroanum_Z8248
C. berteroanum_L10059
C. canescens_T608
C. sp._F195(1)
C. sp._F195
C. tricuspidatum_T2036
C. serrata_M607
C. sp._N2436
C. stuhlmannii_K2307
C. stuhlmannii_Gs.n.(1)
C. stuhlmannii_Gs.n.
C. palustris_K515
C. palustris_K511
C. palustris_WD073
C. sp._K23087
C. palustris_B4389
C. bahiensis_A8731
C. castaneifolia_K778
C. castaneifolia_K1042
C. cubana_G25237
C. heteropetala_K1028
C. heteropetala_K1043
C.sp._V788
C. cordata_A584
C. heteropetala_K1240
C. chiltepecensis_L557
C. cordata_Ss.n.
C. corchoroides_Z1458
A. brandgeei_R1510
A. sp._30013
A. oaxaca_S2974
A. cinerea_S971
P. brasiliensis_Zs.n.
P. brasiliensis_K1046
P. brasiliensis_K1213
P. brasiliensis_W25536
B. dichotoma_C5374
B. dichotoma_J1001
P. penninervia_C22458
P. volubilis_A38
E. disjuncta_N107
S. triandra_G13381
Chiropetalum
Caperonia
Adelia
Philyra
Bernardia
Plukenetia
Enriquebeltrania
Seidelia

Figure S7: Consensus tree of the nuclear markers (*tITE+ETS*) obtained by Bayesian Inference performed in MrBayes. Numbers indicate Bayesian posterior probabilities.

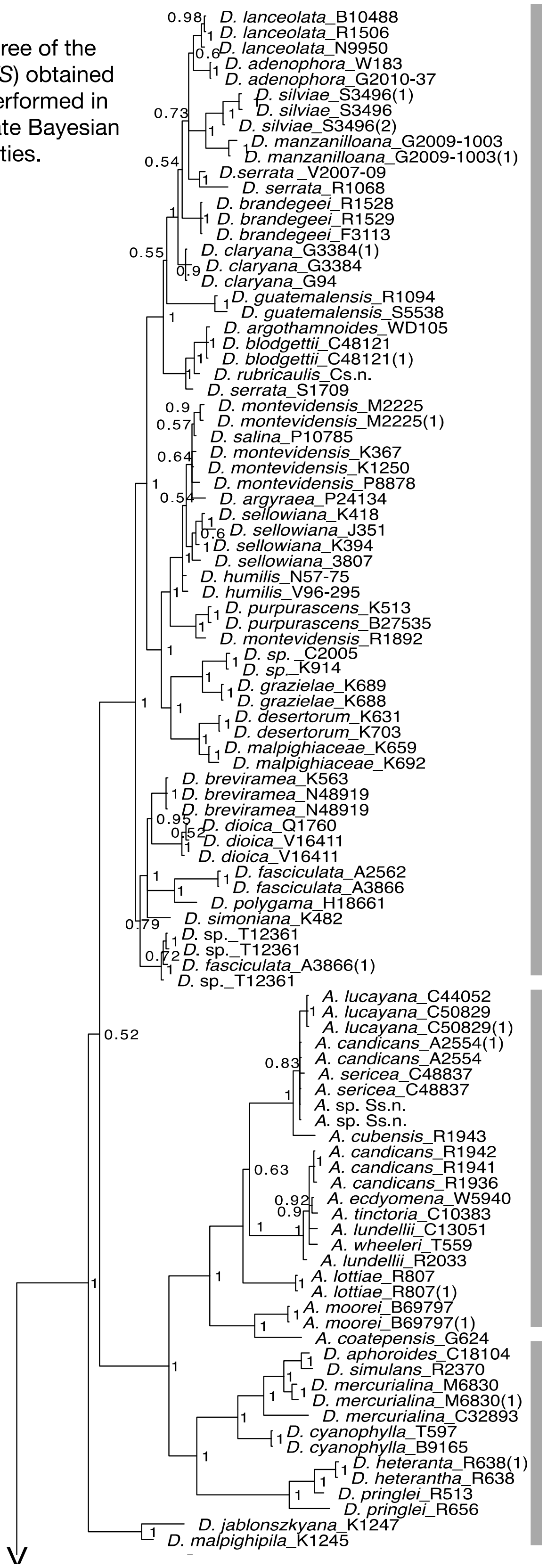

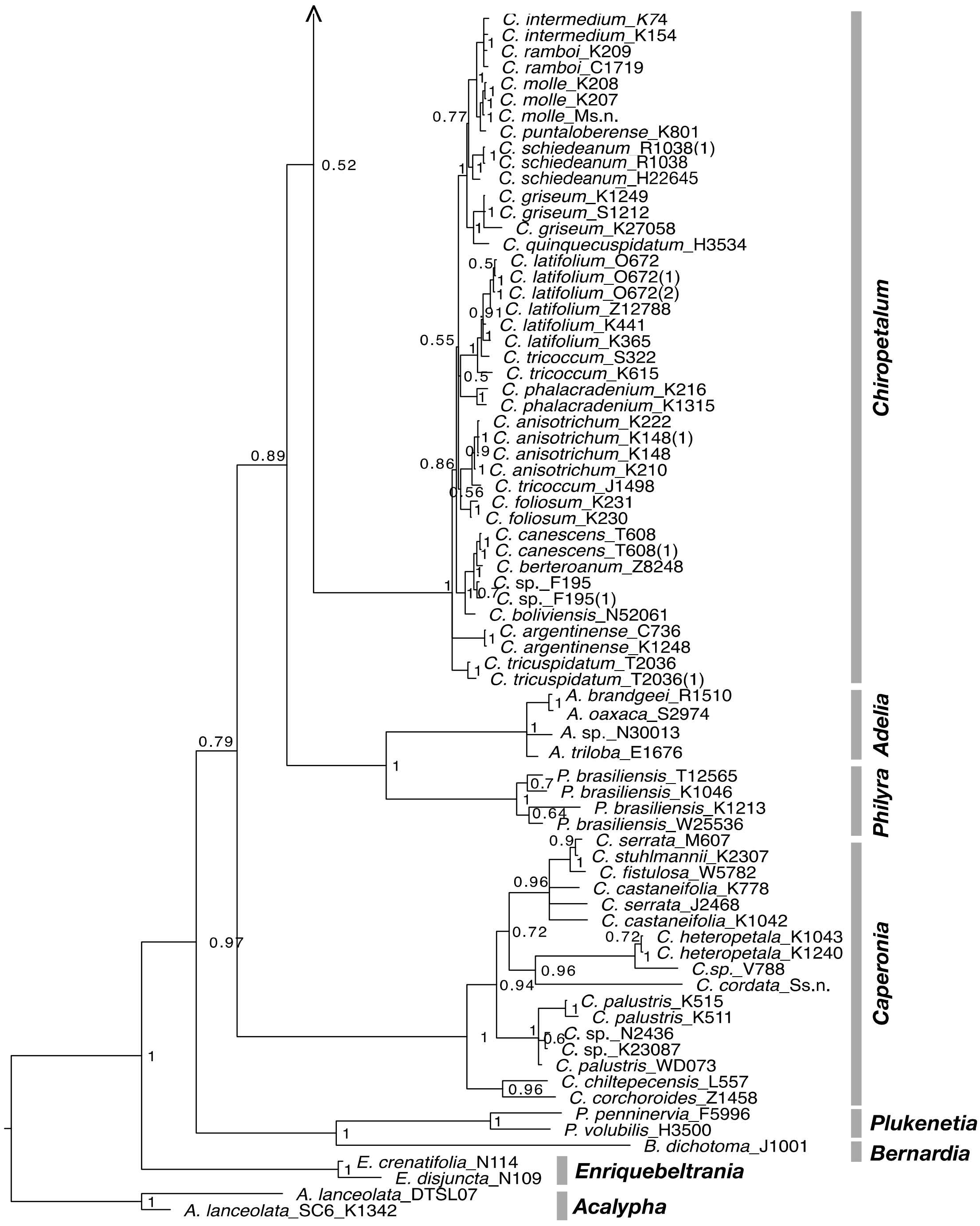

C. intermedium_K74
C. intermedium_K154
C. ramboi_K209
C. ramboi_C1719
C. molle_K208
C. molle_K207
C. molle_Ms.n.
C. puntaloberense_K801
C. schiedeanum_R1038(1)
C. schiedeanum_R1038
C. schiedeanum_H22645
C. griseum_K1249
C. griseum_S1212
C. griseum_K27058
C. quinquecuspidatum_H3534
C. latifolium_O672
C. latifolium_O672(1)
C. latifolium_O672(2)
C. latifolium_Z12788
C. latifolium_K441
C. latifolium_K365
C. tricoccum_S322
C. tricoccum_K615
C. phalacradenium_K216
C. phalacradenium_K1315
C. anisotrichum_K222
C. anisotrichum_K148(1)
C. anisotrichum_K148
C. anisotrichum_K210
C. tricoccum_J1498
C. foliosum_K231
C. foliosum_K230
C. canescens_T608
C. canescens_T608(1)
C. berteroanum_Z8248
C. sp._F195
C. sp._F195(1)
C. boliviensis_N52061
C. argentinense_C736
C. argentinense_K1248
C. tricuspidatum_T2036
C. tricuspidatum_T2036(1)
A. brandgeei_R1510
A. oaxaca_S2974
A. sp._N30013
A. triloba_E1676
P. brasiliensis_T12565
P. brasiliensis_K1046
P. brasiliensis_K1213
P. brasiliensis_W25536
C. serrata_M607
C. stuhlmannii_K2307
C. fistulosa_W5782
C. castaneifolia_K778
C. serrata_J2468
C. castaneifolia_K1042
C. heteropetala_K1043
C. heteropetala_K1240
C.sp._V788
C. cordata_Ss.n.
C. palustris_K515
C. palustris_K511
C. sp._N2436
C. sp._K23087
C. palustris_WD073
C. chiltepecensis_L557
C. corchoroides_Z1458
P. penninervia_F5996
P. volubilis_H3500
B. dichotoma_J1001
E. crenatifolia_N114
E. disjuncta_N109
A. lanceolata_DTSL07
A. lanceolata_SC6_K1342
Chiropetalum
Adelia
Philyra
Caperonia
Plukenetia
Bernardia
Enriquebeltrania
Acalypha
0.52
0.89
0.79
0.97
0.77
0.55
0.86
0.96
0.72
0.94

Figure S8: Consensus tree of all five markers combined obtained by Bayesian Inference performed in MrBayes. Numbers indicate Bayesian posterior probabilities.

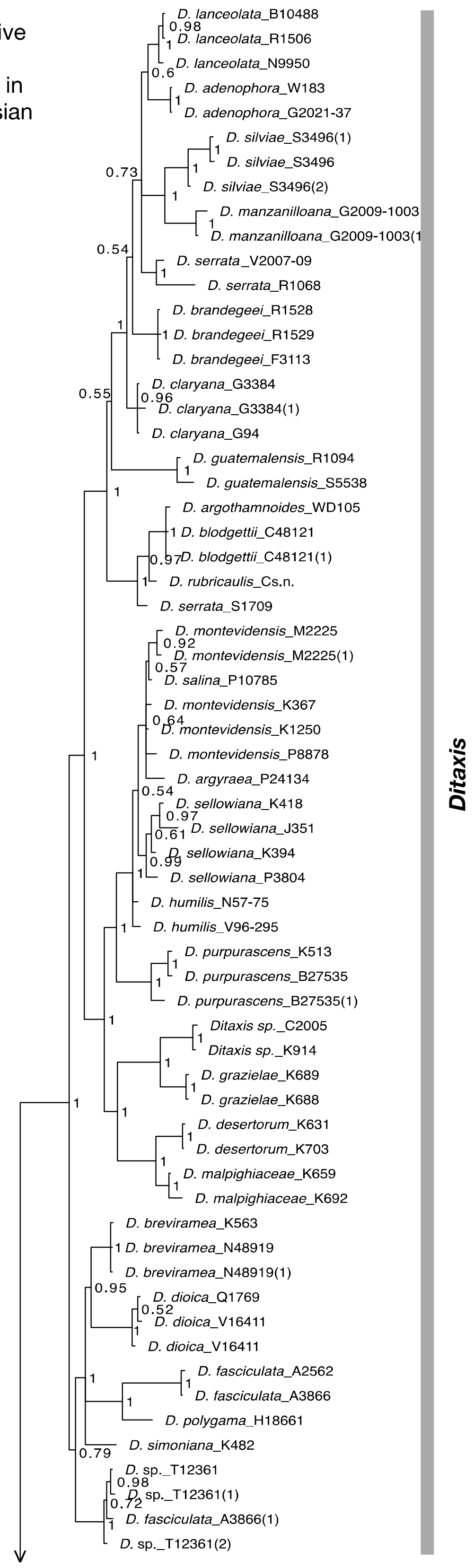

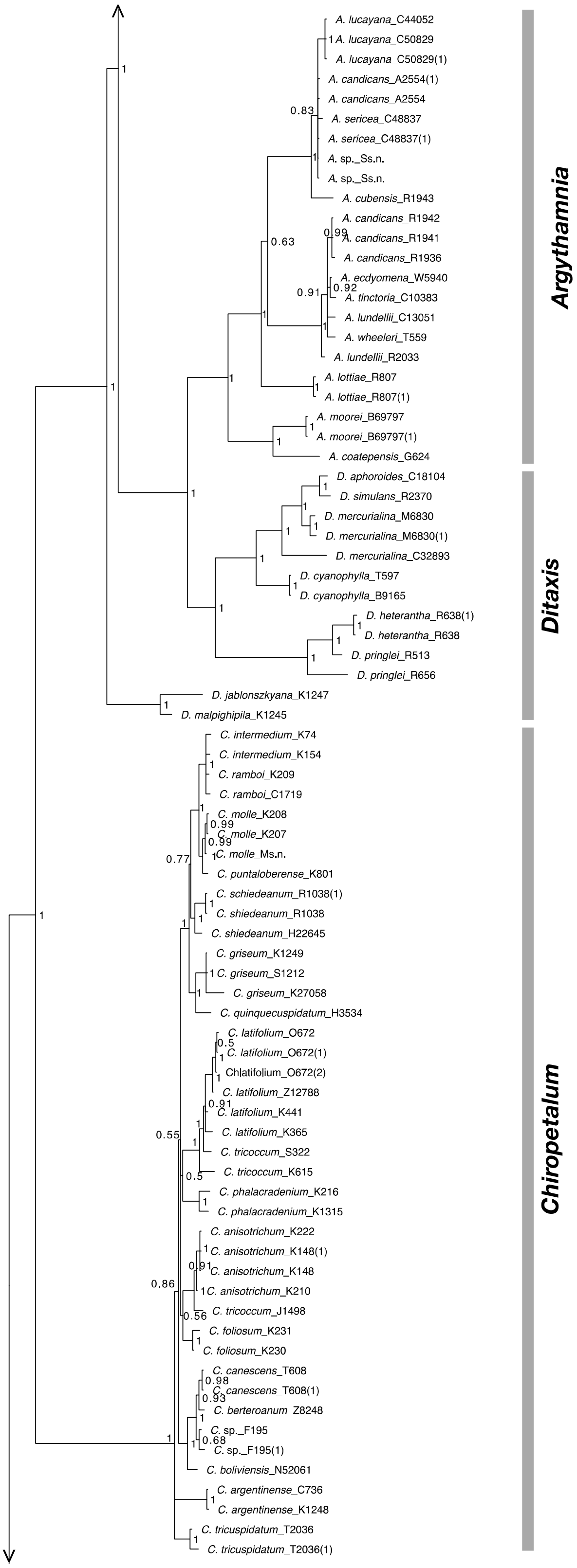

A. lucayana_C44052
A. lucayana_C50829
A. lucayana_C50829(1)
A. candicans_A2554(1)
A. candicans_A2554
A. sericea_C48837
A. sericea_C48837(1)
A. sp._Ss.n.
A. sp._Ss.n.
A. cubensis_R1943
A. candicans_R1942
A. candicans_R1941
A. candicans_R1936
A. ecdyomena_W5940
A. tinctoria_C10383
A. lundellii_C13051
A. wheeleri_T559
A. lundellii_R2033
A. lottiae_R807
A. lottiae_R807(1)
A. moorei_B69797
A. moorei_B69797(1)
A. coatepensis_G624
D. aphoroides_C18104
D. simulans_R2370
D. mercurialina_M6830
D. mercurialina_M6830(1)
D. mercurialina_C32893
D. cyanophylla_T597
D. cyanophylla_B9165
D. heterantha_R638(1)
D. heterantha_R638
D. pringlei_R513
D. pringlei_R656
D. jablonszkyana_K1247
D. malpighipila_K1245
C. intermedium_K74
C. intermedium_K154
C. ramboi_K209
C. ramboi_C1719
C. molle_K208
C. molle_K207
C. molle_Ms.n.
C. puntaloberense_K801
C. schiedeanum_R1038(1)
C. shiedeanum_R1038
C. shiedeanum_H22645
C. griseum_K1249
C. griseum_S1212
C. griseum_K27058
C. quinquecuspidatum_H3534
C. latifolium_O672
C. latifolium_O672(1)
Chlatifolium_O672(2)
C. latifolium_Z12788
C. latifolium_K441
C. latifolium_K365
C. tricoccum_S322
C. tricoccum_K615
C. phalacradenium_K216
C. phalacradenium_K1315
C. anisotrichum_K222
C. anisotrichum_K148(1)
C. anisotrichum_K148
C. anisotrichum_K210
C. tricoccum_J1498
C. foliosum_K231
C. foliosum_K230
C. canescens_T608
C. canescens_T608(1)
C. berteroanum_Z8248
C. sp._F195
C. sp._F195(1)
C. boliviensis_N52061
C. argentinense_C736
C. argentinense_K1248
C. tricuspidatum_T2036
C. tricuspidatum_T2036(1)
Argythamnia
Ditaxis
Chiropetalum

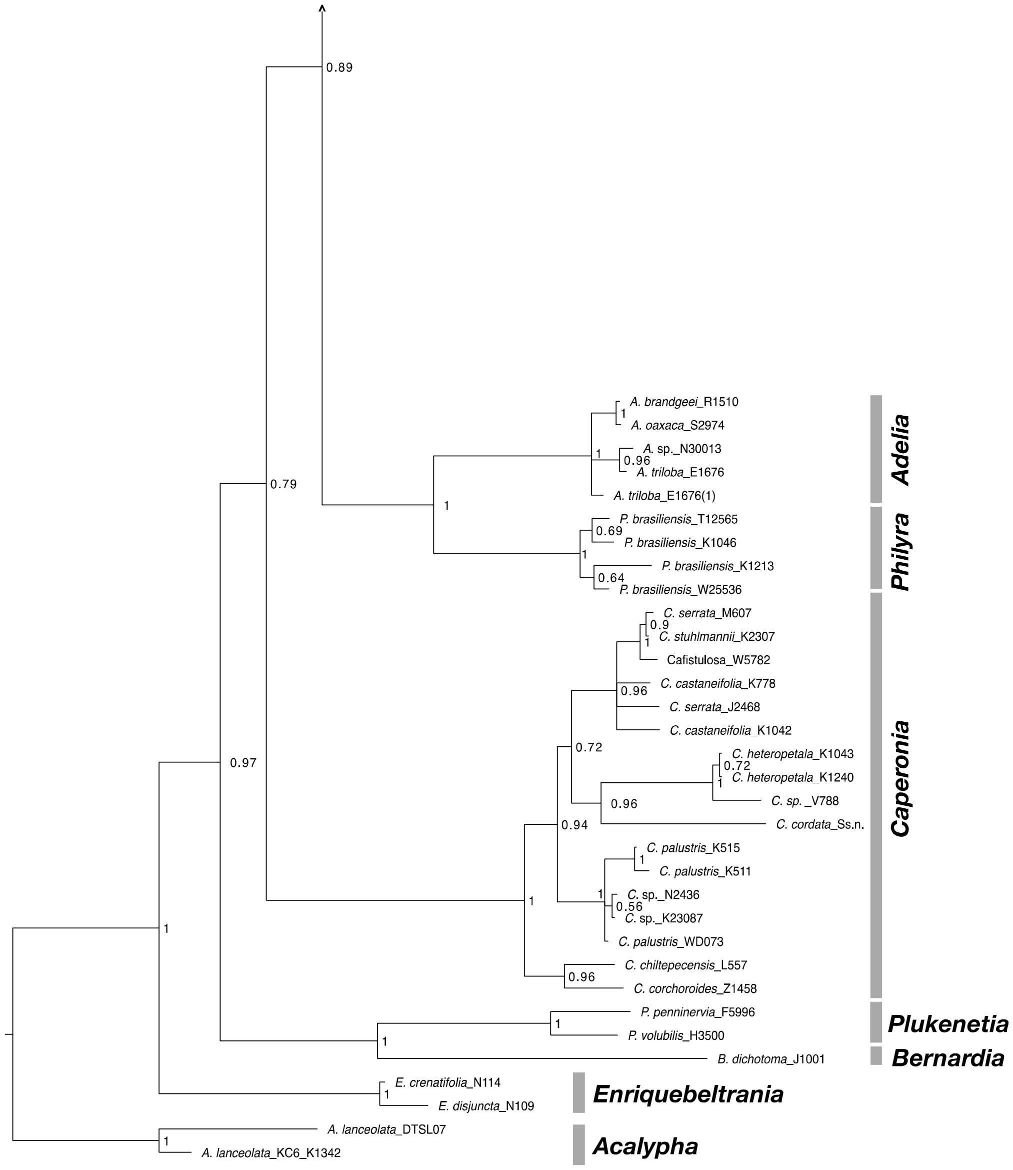

0.89
0.79
0.97
1
A. brandgeei_R1510
A. oaxaca_S2974
A. sp._N30013
A. triloba_E1676
A. triloba_E1676(1)
0.96
P. brasiliensis_T12565
P. brasiliensis_K1046
P. brasiliensis_K1213
P. brasiliensis_W25536
0.69
0.64
C. serrata_M607
C. stuhlmannii_K2307
Cafistulosa_W5782
C. castaneifolia_K778
C. serrata_J2468
C. castaneifolia_K1042
C. heteropetala_K1043
C. heteropetala_K1240
C. sp. _V788
C. cordata_Ss.n.
C. palustris_K515
C. palustris_K511
C. sp._N2436
C. sp._K23087
C. palustris_WD073
C. chiltepecensis_L557
C. corchoroides_Z1458
0.9
0.72
0.94
0.56
P. penninervia_F5996
P. volubilis_H3500
B. dichotoma_J1001
E. crenatifolia_N114
E. disjuncta_N109
A. lanceolata_DTSL07
A. lanceolata_KC6_K1342
Adelia
Philyra
Caperonia
Plukenetia
Bernardia
Enriquebeltrania
Acalypha

Figure S9: Phylogram of all markers combined data set resulting from the RaxML analysis. Numbers indicate the bootstrap support.

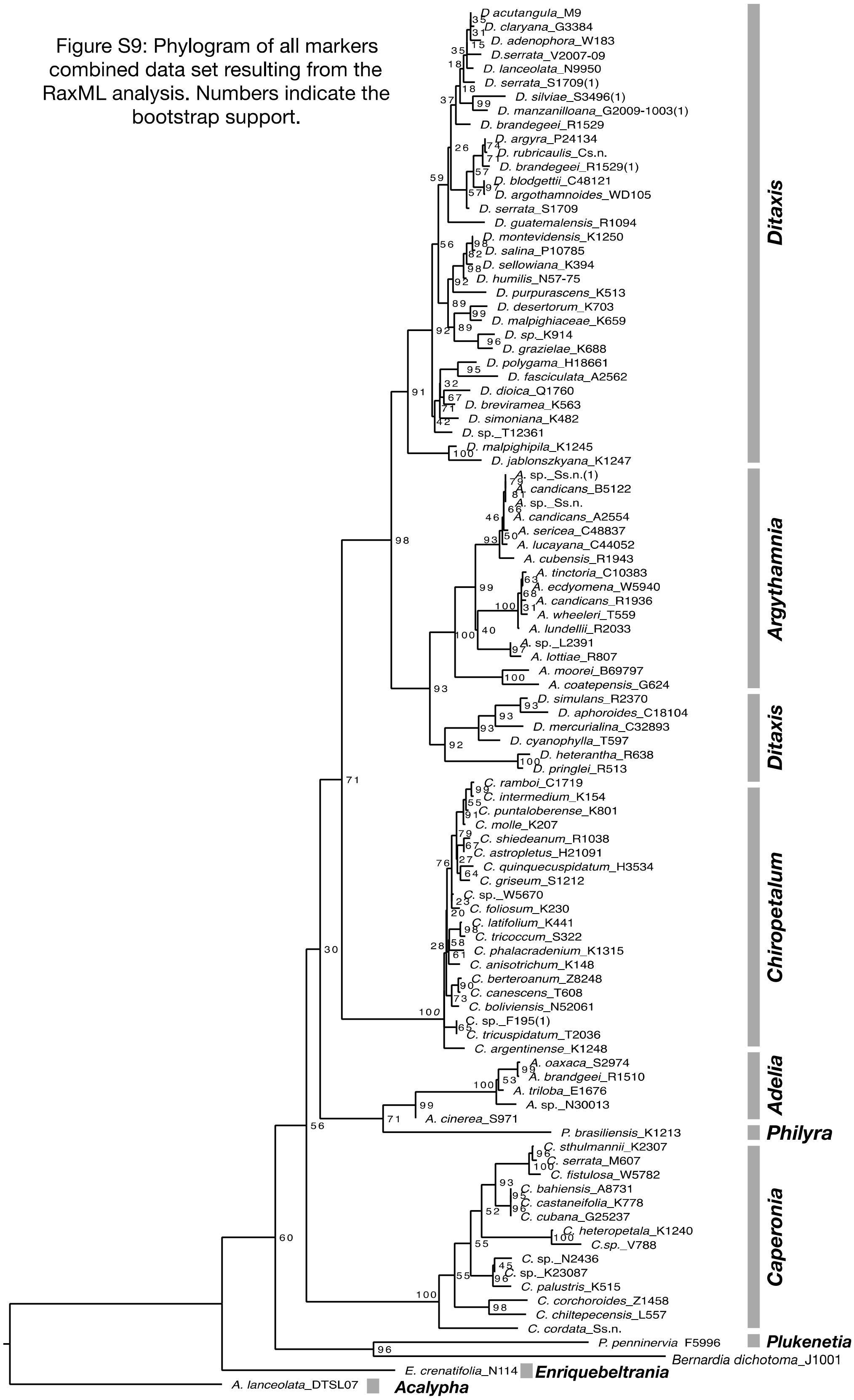

Supplement: Supplementary file 1 [file biology-12-00173-s001.zip › Supplementary material/Supplementary File S2.pdf]
