# Supplementary material for: Systematics of Ditaxinae and Related Lineages within the Subfamily Acalyphoideae (Euphorbiaceae) Based on Molecular Phylogenetics
Source: Biology (Basel). 2023 Jan 21;12(2):173. doi: 10.3390/biology12020173 (PMC9952443; doi:10.3390/biology12020173)
Supplement: Supplementary file 1 [file biology-12-00173-s001.zip › Supplementary material/Supplementary File S3.pdf]

SUPPLEMENTARY MATERIAL S3

Systematics of Ditaxinae and related lineages within subfamily Acalyphoideae (Euphorbiaceae) based on molecular phylogenetics

Josimar Külkamp, Ricarda Riina, Yocupitzia Ramírez-Amezcua, João R.V. Iganci, Inês Cordeiro, Raquel González-Páramo, Sabina Irene Lara-Cabrera and José Fernando A. Baumgratz

Supplementary material S3

Table S1: Summary of the new classification of Ditaxinae and related groups within Acalyphoideae (Euphorbiaceae) and the previous classification. The new classification does not include the subtribe level as in the case of Webster's (2014) scheme.

**New classification** (Külkamp et al.)		**Previous classification** (Webster 2014)		
**Species**	**Tribe**	**Species**	**Subtribe**	**Tribe**
Adelia barbinervis Cham. & Schltdl.	Adelieae	*Adelia barbinervis* Cham. & Schltdl.	-	Adelieae
Adelia brandegeei V.W. Steinm.	Adelieae	*Adelia brandegeei* V.W. Steinm.	-	Adelieae
Adelia cinerea (Wiggins & Rollins) A.Cerv., V.W.Steinm. & Flores-Olvera	Adelieae	*Adelia cinerea* (Wiggins & Rollins) A.Cerv., V.W.Steinm. & Flores-Olvera	-	Adelieae
Adelia membranifolia (Müll.Arg.) Chodat & Hassl.	Adelieae	*Adelia membranifolia* (Müll.Arg.) Chodat & Hassl.	-	Adelieae
Adelia oaxacana (Müll.Arg.) Hemsl.	Adelieae	*Adelia oaxacana* (Müll.Arg.) Hemsl.	-	Adelieae
Adelia obovata Wiggins & Rollins	Adelieae	*Adelia obovata* Wiggins & Rollins	-	Adelieae
Adelia ricinella L.	Adelieae	*Adelia ricinella* L.	-	Adelieae
Adelia triloba (Müll.Arg.) Hemsl.	Adelieae	*Adelia triloba* (Müll.Arg.) Hemsl.	-	Adelieae
Adelia vaseyi (J.M.Coult.) Pax & K.Hoffm.	Adelieae	*Adelia vaseyi* (J.M.Coult.) Pax & K.Hoffm.	-	Adelieae
Argythamnia argyraea Cory	Ditaxeae	*Argythamnia argyraea* Cory	Ditaxinae	Chrozophoreae
Argythamnia arlynniana J.W.Ingram	Ditaxeae	*Ditaxis arlynniana* (J.W.Ingram) Radcl.-Sm. & Govaerts	Ditaxinae	Chrozophoreae
<u>*Argythamnia brandegeei*</u> Millsp.	Ditaxeae	*Ditaxis brandegeei* (Millsp.) Rose & Standl.	Ditaxinae	Chrozophoreae
Argythamnia breviramea Müll.Arg	Ditaxeae	*Ditaxis breviramea* (Müll.Arg.) Pax & K.Hoffm.	Ditaxinae	Chrozophoreae
Argythamnia calycina Müll.Arg.	Ditaxeae	*Ditaxis calycina* (Müll.Arg.) Pax & K.Hoffm.	Ditaxinae	Chrozophoreae
Argythamnia candicans Swartz	Ditaxeae	*Argythamnia candicans* Swartz	Ditaxinae	Chrozophoreae
Argythamnia claryana Jeps.	Ditaxeae	*Ditaxis claryana* (Jeps.) G.L.Webster	Ditaxinae	Chrozophoreae
Argythamnia coatepensis (T.S.Brandegee) Croizat	Ditaxeae	*Argythamnia coatepensis* (T.S.Brandegee) Croizat	Ditaxinae	Chrozophoreae
Argythamnia cubensis Britton & Wilson	Ditaxeae	*Argythamnia cubensis* Britton & Wilson	Ditaxinae	Chrozophoreae
Argythamnia cuneifolia (Pax & K.Hoffm.) J.W.Ingram	Ditaxeae	*Ditaxis cuneifolia* Pax & K.Hoffm.	Ditaxinae	Chrozophoreae
Argythamnia cyanophylla (Wooton & Standl.)	Ditaxeae	*Ditaxis cyanophylla* Wooton & Standl.	Ditaxinae	Chrozophoreae

J.W.Ingram				
Argythamnia depressa (Greenm.) J.W.Ingram	Ditaxeae	*Ditaxis depressa* (Greenm.) Pax & K.Hoffm.	Ditaxinae	Chrozophoreae
Argythamnia desertorum Müll.Arg.	Ditaxeae	*Ditaxis desertorum* (Müll.Arg.) Pax & K.Hoffm.	Ditaxinae	Chrozophoreae
Argythamnia dioica (Bonpland, Humboldt & Kunth) Müll.Arg.	Ditaxeae	*Ditaxis dioica* Bonpland, Humboldt & Kunth	Ditaxinae	Chrozophoreae
Argythamnia ecdyomena J.Ingram	Ditaxeae	*Argythamnia ecdyomena* J.Ingram	Ditaxinae	Chrozophoreae
Argythamnia erubescens J.R.Johnst.	Ditaxeae	*Ditaxis erubescens* (J.R.Johnst.) Pax & K.Hoffm.	Ditaxinae	Chrozophoreae
Argythamnia fasciculata (Vahl ex A.Juss.) Müll.Arg.	Ditaxeae	*Ditaxis fasciculata* Vahl ex A.Juss.	Ditaxinae	Chrozophoreae
Argythamnia fendleri Müll.Arg	Ditaxeae	*Ditaxis fendleri* (Müll.Arg.) Pax & K.Hoffm.	Ditaxinae	Chrozophoreae
Argythamnia grazielae (Külkamp) Külkamp & Riina	Ditaxeae	*Ditaxis grazielae* Külkamp	Ditaxinae	Chrozophoreae
Argythamnia guatemalensis Müll.Arg.	Ditaxeae	*Ditaxis guatemalensis* (Müll.Arg.) Pax & K.Hoffm.	Ditaxinae	Chrozophoreae
Argythamnia haitiensis (Urb.) J.W.Ingram	Ditaxeae	*Argythamnia haitiensis* (Urb.) J.W.Ingram	Ditaxinae	Chrozophoreae
Argythamnia haplostigma Pax & K.Hoffm.	Ditaxeae	*Argythamnia haplostigma* Pax & K.Hoffm.	Ditaxinae	Chrozophoreae
Argythamnia heterantha (Zucc.) Müll.Arg.	Ditaxeae	*Ditaxis heterantha* Zucc.	Ditaxinae	Chrozophoreae
Argythamnia heteropilosa J.Ingram	Ditaxeae	*Argythamnia heteropilosa* J.Ingram	Ditaxinae	Chrozophoreae
Argythamnia humilis (Engelm. & A.Gray) Müll.Arg.	Ditaxeae	*Ditaxis humilis* (Engelm. & A.Gray) Pax	Ditaxinae	Chrozophoreae
Argythamnia illimaniensis (Baill.) Müll.Arg.	Ditaxeae	*Ditaxis illimaniensis* Baill.	Ditaxinae	Chrozophoreae
Argythamnia ingramii Y.Ramírez-Amezcua & V.W.Steinm.	Ditaxeae	*Argythamnia ingramii* Y.Ramírez-Amezcua & V.W.Steinm.	Ditaxinae	Chrozophoreae
Argythamnia jablonszkyana (Pax & K.Hoffm.) J.W.Ingram	Ditaxeae	*Ditaxis jablonszkyana* Pax & K.Hoffm.	Ditaxinae	Chrozophoreae
Argythamnia katharinae (Pax) Croizat	Ditaxeae	*Ditaxis katharinae* Pax	Ditaxinae	Chrozophoreae
Argythamnia lanceolata (Benth.) Müll.Arg.	Ditaxeae	*Ditaxis lanceolata* Benth.	Ditaxinae	Chrozophoreae
Argythamnia lottiae J.Ingram	Ditaxeae	*Argythamnia lottiae* J.Ingram	Ditaxinae	Chrozophoreae
Argythamnia lucayana Millsp.	Ditaxeae	*Argythamnia lucayana* Millsp.	Ditaxinae	Chrozophoreae
Argythamnia lundellii J.Ingram	Ditaxeae	*Argythamnia lundellii* J.Ingram	Ditaxinae	Chrozophoreae
Argythamnia macrantha (Pax & K.Hoffm.) Croizat	Ditaxeae	*Ditaxis macrantha* Pax & K.Hoffm.	Ditaxinae	Chrozophoreae
Argythamnia macrobotrys (Pax & K.Hoffm.) J.W.Ingram	Ditaxeae	*Ditaxis macrobotrys* Pax & K.Hoffm.	Ditaxinae	Chrozophoreae
Argythamnia malpighiacea Ule	Ditaxeae	*Ditaxis malpighiacea* (Ule) Pax & K.Hoffm	Ditaxinae	Chrozophoreae
Argythamnia malpighiphila (Hicken) J.W.Ingram	Ditaxeae	*Ditaxis malpighiphila* (Hicken) L.C.Wheeler	Ditaxinae	Chrozophoreae
Argythamnia manzanilloana Rose	Ditaxeae	*Ditaxis manzanilloana* (Rose) Pax & K.Hoffm.	Ditaxinae	Chrozophoreae
Argythamnia mercurialina (Nutt.) Müll.Arg.	Ditaxeae	*Ditaxis mercurialina* (Nutt.) J.M.Coult.	Ditaxinae	Chrozophoreae
Argythamnia microphylla Pax	Ditaxeae	*Argythamnia microphylla* Pax	Ditaxinae	Chrozophoreae
Argythamnia montevidensis	Ditaxeae	*Ditaxis montevidensis* (Didr.)	Ditaxinae	Chrozophoreae

(Didr.) Müll.Arg.		Pax		
Argythamnia moorei J.Ingram	Ditaxeae	*Argythamnia moorei* J.Ingram	Ditaxinae	Chrozophoreae
Argythamnia oblongifolia Urb.	Ditaxeae	*Argythamnia oblongifolia* Urb.	Ditaxinae	Chrozophoreae
Argythamnia pilosissima (Benth.) Müll.Arg.	Ditaxeae	*Ditaxis pilosissima* (Benth.) A.Heller	Ditaxinae	Chrozophoreae
Argythamnia polygama (Jacq.) Kuntze	Ditaxeae	*Ditaxis polygama* Jacq.	Ditaxinae	Chrozophoreae
Argythamnia pringlei Greenm.	Ditaxeae	*Ditaxis pringlei* (Greenm.) Pax & K.Hoffm.	Ditaxinae	Chrozophoreae
Argythamia proctorii J.Ingram	Ditaxeae	*Argythamia proctorii* J.Ingram	Ditaxinae	Chrozophoreae
Argythamnia purpurascens S.Moore	Ditaxeae	*Ditaxis purpurascens* (S. Moore) Pax & K.Hoffm.	Ditaxinae	Chrozophoreae
Argythamnia rubricaulis (Pax & K.Hoffm.) Croizat	Ditaxeae	*Ditaxis rubricaulis* Pax & K.Hoffm.	Ditaxinae	Chrozophoreae
Argythamnia salina (Pax & K.Hoffm.) J.W.Ingram	Ditaxeae	*Ditaxis salina* Pax & K.Hoffm.	Ditaxinae	Chrozophoreae
Argythamnia sellowiana (Pax & K.Hoffm.) J.W.Ingram	Ditaxeae	*Ditaxis sellowiana* Pax & K.Hoffm.	Ditaxinae	Chrozophoreae
Argythamnia sericea Griseb.	Ditaxeae	*Argythamnia sericea* Griseb.	Ditaxinae	Chrozophoreae
Argythamnia serrata (Torr.) Müll.Arg.	Ditaxeae	*Ditaxis serrata* (Torr.) A.Heller	Ditaxinae	Chrozophoreae
Argythamnia silviae Y.Ramírez-Amezcua & V.W.Steinm.	Ditaxeae	*Argythamnia silviae* Y.Ramírez-Amezcua & V.W.Steinm.	Ditaxinae	Chrozophoreae
Argythamnia simoniana (Casar.) Müll.Arg.	Ditaxeae	*Ditaxis simoniana* Casar.	Ditaxinae	Chrozophoreae
Argythamnia simulans J.W.Ingram	Ditaxeae	*Argythamnia simulans* J.W.Ingram	Ditaxinae	Chrozophoreae
Argythamnia sitiens (T.S.Brandegee) J.Ingram	Ditaxeae	*Argythamnia sitiens* (T.S.Brandegee) J.Ingram	Ditaxinae	Chrozophoreae
Argythamnia stahlii Urb.	Ditaxeae	*Argythamnia stahlii* Urb.	Ditaxinae	Chrozophoreae
Argythamnia tinctoria Mill.	Ditaxeae	*Argythamnia tinctoria* Mill.	Ditaxinae	Chrozophoreae
Argythamnia wheeleri J.Ingram	Ditaxeae	*Argythamnia wheeleri* J.Ingram	Ditaxinae	Chrozophoreae
Caperonia aculeolata Müll.Arg.	Caperonieae	*Caperonia aculeolata* Müll.Arg.	Ditaxinae	Chrozophoreae
Caperonia altissima Eskuche	Caperonieae	*Caperonia altissima* Eskuche	Ditaxinae	Chrozophoreae
Caperonia amarumayu Külkamp & Cordeiro	Caperonieae	*Caperonia amarumayu* Külkamp & Cordeiro	Ditaxinae	Chrozophoreae
Caperonia angustissima Klotzsch	Caperonieae	*Caperonia angustissima* Klotzsch	Ditaxinae	Chrozophoreae
Caperonia bahiensis Müll.Arg.	Caperonieae	*Caperonia bahiensis* Müll.Arg.	Ditaxinae	Chrozophoreae
Caperonia buettneriacea Müll.Arg.	Caperonieae	*Caperonia buettneriacea* Müll.Arg.	Ditaxinae	Chrozophoreae
Caperonia capiibariensis Eskuche	Caperonieae	*Caperonia capiibariensis* Eskuche	Ditaxinae	Chrozophoreae
Caperonia castaneifolia (L.) A.St.-Hil.	Caperonieae	*Caperonia castaneifolia* (L.) A.St.-Hil.	Ditaxinae	Chrozophoreae
Caperonia castrobarrosiana Paula & Hamburgo	Caperonieae	*Caperonia castrobarrosiana* Paula & Hamburgo	Ditaxinae	Chrozophoreae
Caperonia chiltepecensis Croizat	Caperonieae	*Caperonia chiltepecensis* Croizat	Ditaxinae	Chrozophoreae
Caperonia corchoroides Müll.Arg.	Caperonieae	*Caperonia corchoroides* Müll.Arg.	Ditaxinae	Chrozophoreae
Caperonia cordata A.St.-Hil.	Caperonieae	*Caperonia cordata* A.St.-Hil.	Ditaxinae	Chrozophoreae
Caperonia cubana Pax & K.Hoffm.	Caperonieae	*Caperonia cubana* Pax & K.Hoffm.	Ditaxinae	Chrozophoreae

Caperonia fistulosa Beille	Caperonieae	*Caperonia fistulosa* Beille	Ditaxinae	Chrozophoreae
Caperonia gardneri Müll.Arg.	Caperonieae	*Caperonia gardneri* Müll.Arg.	Ditaxinae	Chrozophoreae
Caperonia glabrata Pax & K.Hoffm.	Caperonieae	*Caperonia glabrata* Pax & K.Hoffm.	Ditaxinae	Chrozophoreae
Caperonia heteropetala Didr.	Caperonieae	*Caperonia heteropetala* Didr.	Ditaxinae	Chrozophoreae
Caperonia hystrix Pax & K.Hoffm.	Caperonieae	*Caperonia hystrix* Pax & K.Hoffm.	Ditaxinae	Chrozophoreae
Caperonia langsdorffii Müll.Arg.	Caperonieae	*Caperonia langsdorffii* Müll.Arg.	Ditaxinae	Chrozophoreae
Caperonia latifolia Pax	Caperonieae	*Caperonia latifolia* Pax	Ditaxinae	Chrozophoreae
Caperonia linearifolia A.St.-Hil.	Caperonieae	*Caperonia linearifolia* A.St.-Hil.	Ditaxinae	Chrozophoreae
Caperonia lutea Pax & K.Hoffm.	Caperonieae	*Caperonia lutea* Pax & K.Hoffm.	Ditaxinae	Chrozophoreae
Caperonia maracaibensis Külkamp & Cordeiro	Caperonieae	*Caperonia maracaibensis* Külkamp & Cordeiro	Ditaxinae	Chrozophoreae
Caperonia multicostata Müll.Arg.	Caperonieae	*Caperonia multicostata* Müll.Arg.	Ditaxinae	Chrozophoreae
Caperonia neglecta G.L.Webster	Caperonieae	*Caperonia neglecta* G.L.Webster	Ditaxinae	Chrozophoreae
Caperonia palustris (L.) A.St.-Hil.	Caperonieae	*Caperonia palustris* (L.) A.St.-Hil.	Ditaxinae	Chrozophoreae
Caperonia paraguayensis Pax & K.Hoffm.	Caperonieae	*Caperonia paraguayensis* Pax & K.Hoffm.	Ditaxinae	Chrozophoreae
Caperonia regnellii Müll.Arg.	Caperonieae	*Caperonia regnellii* Müll.Arg.	Ditaxinae	Chrozophoreae
Caperonia rutenbergii Müll.Arg.	Caperonieae	*Caperonia rutenbergii* Müll.Arg.	Ditaxinae	Chrozophoreae
Caperonia serrata (Turcz.) C.Presl.	Caperonieae	*Caperonia serrata* (Turcz.) C.Presl.	Ditaxinae	Chrozophoreae
Caperonia similis Pax & K.Hoffm.	Caperonieae	*Caperonia similis* Pax & K.Hoffm.	Ditaxinae	Chrozophoreae
Caperonia stenophylla Müll.Arg.	Caperonieae	Caperonia stenophylla Müll.Arg.	Ditaxinae	Chrozophoreae
Caperonia stuhlmannii Pax	Caperonieae	*Caperonia stuhlmannii* Pax	Ditaxinae	Chrozophoreae
Caperonia subrotunda Chiov.	Caperonieae	*Caperonia subrotunda* Chiov.	Ditaxinae	Chrozophoreae
Caperonia zaponzeta Mansf.	Caperonieae	*Caperonia zaponzeta* Mansf.	Ditaxinae	Chrozophoreae
Chiropetalum anisotrichum (Müll.Arg.) Pax & K.Hoffm.	Ditaxeae	*Chiropetalum anisotrichum* (Müll.Arg.) Pax & K.Hoffm.	Ditaxinae	Chrozophoreae
Chiropetalum argentinense Skottsb.	Ditaxeae	*Chiropetalum argentinense* Skottsb.	Ditaxinae	Chrozophoreae
Chiropetalum astroplethos (J.W. Ingram) Radcl.-Sm. & Govaerts	Ditaxeae	*Chiropetalum astroplethos* (J.W. Ingram) Radcl.-Sm. & Govaerts	Ditaxinae	Chrozophoreae
Chiropetalum berteroanum Schltdl.	Ditaxeae	*Chiropetalum berteroanum* Schltdl.	Ditaxinae	Chrozophoreae
Chiropetalum boliviense (Müll.Arg.) Pax & K.Hoffm.	Ditaxeae	*Chiropetalum boliviense* (Müll.Arg.) Pax & K.Hoffm.	Ditaxinae	Chrozophoreae
Chiropetalum canescens Phil.	Ditaxeae	*Chiropetalum canescens* Phil.	Ditaxinae	Chrozophoreae
Chiropetalum cremnophilum I.M.Johnst.	Ditaxeae	*Chiropetalum cremnophilum* I.M.Johnst.	Ditaxinae	Chrozophoreae
Chiropetalum foliosum Pax & K.Hoffm.	Ditaxeae	*Chiropetalum foliosum* Pax & K.Hoffm.	Ditaxinae	Chrozophoreae
Chiropetalum griseum Griseb.	Ditaxeae	*Chiropetalum griseum* Griseb.	Ditaxinae	Chrozophoreae
Chiropetalum intermedium Pax & K.Hoffm.	Ditaxeae	*Chiropetalum intermedium* Pax & K.Hoffm.	Ditaxinae	Chrozophoreae
Chiropetalum molle (Klotzsch ex. Baill.) Pax & K.Hoffm.	Ditaxeae	*Chiropetalum molle* (Klotzsch ex. Baill.) Pax & K.Hoffm.	Ditaxinae	Chrozophoreae

Chiropetalum patagonicum (Speg.) O'Donell & Lourteig	Ditaxeae	*Chiropetalum patagonicum* (Speg.) O'Donell & Lourteig	Ditaxinae	Chrozophoreae
Chiropetalum pavonianum (Müll.Arg.) Pax	Ditaxeae	*Chiropetalum pavonianum* (Müll.Arg.) Pax	Ditaxinae	Chrozophoreae
Chiropetalum phalacradenium (J.W.Ingram) L.B.Sm. & Downs	Ditaxeae	*Chiropetalum phalacradenium* (J.W.Ingram) L.B.Sm. & Downs	Ditaxinae	Chrozophoreae
Chiropetalum puntaloberense Alonso Paz & Bassagoda	Ditaxeae	*Chiropetalum puntaloberense* Alonso Paz & Bassagoda	Ditaxinae	Chrozophoreae
Chiropetalum quinquecuspidatum (A.Juss.) Pax & K.Hoffm.	Ditaxeae	*Chiropetalum quinquecuspidatum* (A.Juss.) Pax & K.Hoffm.	Ditaxinae	Chrozophoreae
Chiropetalum ramboi (Allem & Irgang) Radcl.-Sm. & Govaerts	Ditaxeae	*Chiropetalum ramboi* (Allem & Irgang) Radcl.-Sm. & Govaerts	Ditaxinae	Chrozophoreae
Chiropetalum ruizianum (Müll.Arg.) Pax & K.Hoffm.	Ditaxeae	*Chiropetalum ruizianum* (Müll.Arg.) Pax & K.Hoffm.	Ditaxinae	Chrozophoreae
Chiropetalum schiedeanum (Müll.Arg.) Pax	Ditaxeae	*Chiropetalum schiedeanum* (Müll.Arg.) Pax	Ditaxinae	Chrozophoreae
Chiropetalum tricoccum (Vell.) Chodat & Hassl	Ditaxeae	*Chiropetalum tricoccum* (Vell.) Chodat & Hassl	Ditaxinae	Chrozophoreae
Chiropetalum tricuspidatum (Lam.) A.Juss.	Ditaxeae	*Chiropetalum tricuspidatum* (Lam.) A.Juss.	Ditaxinae	Chrozophoreae
Garciadelia abbottii Jestrow & Jiménez Rodr.	Adelieae	*Garciadelia abbottii* Jestrow & Jiménez Rodr.	-	Adelieae
Garciadelia castilloae Jestrow & Jiménez Rodr.	Adelieae	*Garciadelia castilloae* Jestrow & Jiménez Rodr.	-	Adelieae
Garciadelia leprosa (Willd.) Jestrow & Jiménez Rodr.	Adelieae	*Garciadelia leprosa* (Willd.) Jestrow & Jiménez Rodr.	-	Adelieae
Garciadelia mejiae Jestrow & Jiménez Rodr.	Adelieae	*Garciadelia mejiae* Jestrow & Jiménez Rodr.	-	Adelieae
Lasiocroton bahamensis Pax & K.Hoffm.	Adelieae	*Lasiocroton bahamensis* Pax & K.Hoffm.	-	Adelieae
Lasiocroton fawcettii Urb.	Adelieae	*Lasiocroton fawcettii* Urb.	-	Adelieae
Lasiocroton gracilis Britton & P.Wilson	Adelieae	*Lasiocroton gracilis* Britton & P.Wilson	-	Adelieae
Lasiocroton gutierrezii Jestrow	Adelieae	*Lasiocroton gutierrezii* Jestrow	-	Adelieae
Lasiocroton harrisii Britton	Adelieae	*Lasiocroton harrisii* Britton	-	Adelieae
Lasiocroton macrophyllus (Sw.) Griseb.	Adelieae	*Lasiocroton macrophyllus* (Sw.) Griseb.	-	Adelieae
Lasiocroton microphyllus (A.Rich.) Jestrow	Adelieae	*Lasiocroton microphyllus* (A.Rich.) Jestrow	-	Adelieae
Leucocroton acunae Borhidi	Adelieae	*Leucocroton acunae* Borhidi	-	Adelieae
Leucocroton anomalus Borhidi	Adelieae	*Leucocroton anomalus* Borhidi	-	Adelieae
Leucocroton bracteosus Urb.	Adelieae	*Leucocroton bracteosus* Urb.	-	Adelieae
Leucocroton brittonii Alain	Adelieae	*Leucocroton brittonii* Alain	-	Adelieae
Leucocroton comosus Urb.	Adelieae	*Leucocroton comosus* Urb.	-	Adelieae
Leucocroton cordifolius (Britton & P.Wilson) Alain	Adelieae	*Leucocroton cordifolius* (Britton & P.Wilson) Alain	-	Adelieae
Leucocroton discolor Urb.	Adelieae	*Leucocroton discolor* Urb.	-	Adelieae
Leucocroton ekmanii Urb.	Adelieae	*Leucocroton ekmanii* Urb.	-	Adelieae
Leucocroton flavicans Müll.Arg.	Adelieae	*Leucocroton flavicans* Müll.Arg.	-	Adelieae
Leucocroton havanensis Borhidi	Adelieae	*Leucocroton havanensis* Borhidi	-	Adelieae
Leucocroton incrustatus Borhidi	Adelieae	*Leucocroton incrustatus* Borhidi	-	Adelieae

Leucocroton linearifolius Britton	Adelieae	*Leucocroton linearifolius* Britton	-	Adelieae
Leucocroton longibracteatus Borhidi	Adelieae	*Leucocroton longibracteatus* Borhidi	-	Adelieae
Leucocroton moaensis Borhidi & O.Muñiz	Adelieae	*Leucocroton moaensis* Borhidi & O.Muñiz	-	Adelieae
Leucocroton moncadae Borhidi	Adelieae	*Leucocroton moncadae* Borhidi	-	Adelieae
Leucocroton obovatus Urb.	Adelieae	*Leucocroton obovatus* Urb.	-	Adelieae
Leucocroton pachyphylloides Borhidi	Adelieae	*Leucocroton pachyphylloides* Borhidi	-	Adelieae
Leucocroton pachyphyllus Urb.	Adelieae	*Leucocroton pachyphyllus* Urb.	-	Adelieae
Leucocroton pallidus Britton& P.Wilson	Adelieae	*Leucocroton pallidus* Britton& P.Wilson	-	Adelieae
Leucocroton revolutus C.Wright	Adelieae	*Leucocroton revolutus* C.Wright	-	Adelieae
Leucocroton sameki Borhidi	Adelieae	*Leucocroton sameki* Borhidi	-	Adelieae
Leucocroton saxicola Britton	Adelieae	*Leucocroton saxicola* Britton	-	Adelieae
Leucocroton stenophyllus Urb.	Adelieae	*Leucocroton stenophyllus* Urb.	-	Adelieae
Leucocroton subpeltatus (Urb.) Alain	Adelieae	*Leucocroton subpeltatus* (Urb.) Alain	-	Adelieae
Leucocroton virens Griseb.	Adelieae	*Leucocroton virens* Griseb.	-	Adelieae
Leucocroton wrightii Griseb.	Adelieae	*Leucocroton wrightii* Griseb.	-	Adelieae
Philyra brasiliensis Klotzsch	Adelieae	*Philyra brasiliensis* Klotzsch	-	Adelieae
